# Productive and Penicillin-Stressed *Chlamydia pecorum* Infection Induces Nuclear Factor Kappa B Activation and Interleukin-6 Secretion *In Vitro*

**DOI:** 10.3389/fcimb.2017.00180

**Published:** 2017-05-11

**Authors:** Cory A. Leonard, Robert V. Schoborg, Nicole Borel

**Affiliations:** ^1^Department of Pathobiology, Institute of Veterinary Pathology, University of ZurichZurich, Switzerland; ^2^Department of Biomedical Sciences, Center for Inflammation, Infectious Disease and Immunity, James H. Quillen College of Medicine, East Tennessee State UniversityJohnson City, TN, USA

**Keywords:** *Chlamydia pecorum*, nuclear factor kappa B, interleukin-6, chlamydial persistence, HeLa cells

## Abstract

Nuclear factor kappa B (NFκB) is an inflammatory transcription factor that plays an important role in the host immune response to infection. The potential for chlamydiae to activate NFκB has been an area of interest, however most work has focused on chlamydiae impacting human health. Given that inflammation characteristic of chlamydial infection may be associated with severe disease outcomes or contribute to poor overall fitness in farmed animals, we evaluated the ability of porcine chlamydiae to induce NFκB activation *in vitro*. *C. pecorum* infection induced both NFκB nuclear translocation and activation at 2 hours post infection (hpi), an effect strongly enhanced by suppression of host *de novo* protein synthesis. *C. suis* and *C. trachomatis* showed less capacity for NFκB activation compared to *C. pecorum*, suggesting a species-specific variation in NFκB activation. At 24 hpi, *C. pecorum* induced significant NFκB activation, an effect not abolished by penicillin (beta lactam)-induced chlamydial stress. *C. pecorum*-dependent secretion of interleukin 6 was also detected in the culture supernatant of infected cells at 24 hpi, and this effect, too, was unchanged by penicillin-induced chlamydial stress. Taken together, these results suggest that NFκB participates in the early inflammatory response to *C. pecorum* and that stressed chlamydiae can promote inflammation.

## Introduction

The chlamydiae, obligate intracellular Gram-negative bacterial pathogens, cause a broad range of diseases in non-human animals and humans. All chlamydiae develop inside of a membrane-delimited compartment termed the inclusion and share a characteristic biphasic developmental cycle. Infectious elementary bodies (EBs) adhere to and enter host cells, where they differentiate into replicative, but non-infectious, reticulate bodies (RBs); RBs divide by binary fission and differentiate back into infectious EBs, which exit the cell by lysis or extrusion (Moulder, 1991; Abdelrahman and Belland, 2005; Hybiske and Stephens, 2007). *In vitro*, various stressors, including host immune response factors, nutrient deprivation, beta lactam antibiotic exposure, or co-infection with viruses or parasites, are capable of interrupting RB replicative division and maturation to EBs and inducing aberrant body (AB) formation. Upon removal of the stressor, normal chlamydial development resumes, and eventual release of infectious EBs continues. This divergence from the normal developmental cycle results in a reversible lack of infectivity, termed chlamydial persistence or the chlamydial stress response (Hogan et al., 2004; Wyrick, 2010; Schoborg, 2011; Bavoil, 2014). While some chlamydial forms consistent with AB have been reported in both human (Borel et al., 2008) and animal (Pospischil et al., 2009) tissue samples, and chlamydial persistence has been induced experimentally in mice, wherein it limited the efficacy of subsequent azithromycin treatment (Phillips Campbell et al., 2012; Phillips-Campbell et al., 2014), the biological significance of the chlamydial stress response in natural infections is unknown.

Chlamydiosis encompasses a spectrum of clinical presentations, from asymptomatic infection, to acute and fatal disease, to chronic disease associated with sustained or repeated infection. Chlamydial disease impacts human health globally, in both developed and developing nations. *Chlamydia trachomatis* is the leading cause of infectious blindness (trachoma) and the most common bacterial sexually transmitted infection, worldwide (Global incidence and prevalence of selected curable sexually transmitted infections – 2008; WHO, 2014). In trachoma, eyelid deformation, due to the inflammatory response to chronic or repeated chlamydial infection, leads to inturned lashes that damage the cornea and ultimately cause blindness (Hu et al., 2013). In women, *C. trachomatis* genital infection is capable of ascending to the upper reproductive tract, where chronic inflammation may result in permanent fallopian tube damage, leading to infertility (Hafner, 2015). *Chlamydia pneumoniae*, a highly prevalent agent of respiratory infection, has also been implicated in the development of atherosclerotic plaques and subsequent coronary artery and cerebrovascular disease. In particular, *C. pneumoniae* infected macrophages may release cytokines that promote chronic inflammation in coronary vasculature (Fazio et al., 2009). Thus, inflammation is a common characteristic of the most detrimental clinical outcomes of human chlamydial disease.

Chlamydial infection is widespread in economically important farmed animals and in wild animal populations as well. Chlamydial disease in the koala, for example, caused primarily by *Chlamydia pecorum*, in many ways mirrors *C. trachomatis* disease in humans, causing inflammation leading to blinding ocular disease and urogenital infections associated with reduced fertility and contributing to population decline (Polkinghorne et al., 2013). Chronic chlamydial infections are more common than acute disease outbreaks in agricultural animals, especially swine and ruminants, are likely to be asymptomatic and are frequently endemic. *Chlamydia suis*, the primary chlamydial species infecting pigs, has been reported in >90% of Swiss fattening pigs, and was associated with mild diarrhea but not overt disease (Hoffmann et al., 2015). A range of symptoms, as well as asymptomatic infection, have also been attributed to various chlamydial species in pigs, and such infections are expected to have economic importance (Schautteet and Vanrompay, 2011). Similarly, *C. pecorum, C. abortus, C. psittaci*, and *C. suis* infections are common in cattle, often in the absence of clinical symptoms, but may be associated with lack of productivity or fertility when found in conjunction with various other epidemiological risk factors (Reinhold et al., 2011). For example, *C. pecorum* infections, even when asymptomatic, have been shown to reduce growth rates in calves by almost 50% (Poudel et al., 2012). Therefore, in an agricultural setting, inflammation is also characteristic of chlamydial infection and is not only associated with severe disease outcomes, but may also contribute to poor overall fitness with subsequent negative economic impact.

The inflammatory response, though causative of pathogenesis in chronic chlamydial disease, is, in fact, vital for host defense and resolution of infection. Nuclear factor kappa B (NFκB) is an inflammatory transcription factor that plays an important role in the host immune response to infection. NFκB is expressed not only in immune cells, but also in the epithelial cells initially targeted by *Chlamydia* and other pathogens, where it can elicit normal or aberrant induction of epithelial secretion of inflammatory cytokines, such as interleukin 6 (IL-6) (Alberti et al., 2012; Hoesel and Schmid, 2013). In mammals, the NFκB transcription factor is comprised of a family of five subunits: p65, p50, p52, c-Rel, and Rel B. These subunits form a series of homodimers and heterodimers which target a variety of conserved DNA sequences, known as kappa B binding sites, to elicit both activating and repressing modulation of a broad range of genes involved in biological processes including development, immunity, and oncogenesis (Gilmore, 2006; Hayden et al., 2006). Prior to stimulation, inactive NFκB dimers reside in the cytoplasm, where they are complexed with members of the IkB family (inhibitors of NFκB). Stimulation can be mediated by a variety of receptors, including Toll-like receptors, various interleukin and cytokine receptors, and antigen receptors (Hoesel and Schmid, 2013). Such stimulation results, most typically, in the phosphorylation of IkB by the IKK (IkB kinase) complex, the subsequent proteosomal degradation of IkB, and the translocation of active NFκB to the nucleus (Gilmore, 2006).

NFκB is an integral component in selective activation of the host response to pathogen recognition receptor activation, and is thus an essential element in the subsequent innate and adaptive immune responses (Hayden et al., 2006). As such, the potential for chlamydiae to activate NFκB has been an area of substantial interest, toward the goal of elucidating the mechanisms of the damaging inflammatory nature of chlamydial infection. The majority of this interest has been focused on the chlamydiae most impacting human health, namely *C. pneumoniae* and *C. trachomatis*, with less frequent evaluation of *C. muridarum*, widely used in murine models of chlamydiosis, and zoonotic *C. psittaci*. While *C. pneumoniae* has been consistently reported to induce NFκB activation in macrophages or smooth muscle cells, the reports for *C. trachomatis* have been more variable, from activation of NFκB to chlamydial-dependent degradation of NFκB, with evaluation encompassing a broad array of cells types and experimental conditions. Moreover, the observation that chlamydial protease activity factor (CPAF) degrades various host proteins, including the NFκB p65 subunit, during routine cell lysis and sample processing has further confounded efforts to clarify the role of NFκB in *Chlamydia*-dependent inflammation (Chen et al., 2012; Tan and Sütterlin, 2014; Johnson et al., 2015).

We aimed to evaluate the ability of porcine chlamydiae to induce NFκB activation *in vitro*. *C. pecorum* induced both NFκB nuclear translocation and activation at 2 hours post infection (hpi), an effect strongly enhanced by suppression of host *de novo* protein synthesis. *C. suis*, and the closely related *C. trachomatis* (serovar E), showed much reduced capacity for NFκB activation compared to *C. pecorum*, indicative of species-specific variation in the ability to induce NFκB activation. At 24 hpi, *C. pecorum* induced significant NFκB activation, even in the absence of experimental inhibition of host protein synthesis—an effect not abolished by penicillin (beta lactam)-induced chlamydial stress. Notably, when host protein synthesis was not inhibited, substantial cytoplasmic localization of active NFκB was observed at 24 hpi, similar to recently reported CPAF-dependent prevention of NFκB nuclear translocation in *C. trachomatis* L2 *in vitro* infection (Patton et al., 2016), suggesting a shared inhibitory effect on NFκB function among different chlamydial species. *C. pecorum*-dependent secretion of interleukin 6, a pro-inflammatory cytokine modulated by inflammatory transcription factors including, but not limited to, NFκB was also detected in the culture supernatant of infected cells at 24 hpi, and this effect, too, was unchanged by penicillin-induced stress. Taken together, these results suggest NFκB participates in the early inflammatory response to *C. pecorum*. Additionally, penicillin, heavily used in agriculture, may be expected to induce chlamydial stress, rather than curing chlamydial infection; our data suggest that such stressed chlamydiae may still promote inflammation.

## Materials and methods

### Host cell cultivation and media

HeLa cells are a human cervical adenocarcinoma epithelial cell line used frequently for porcine chlamydiae studies (Lenart et al., 2001; Leonard et al., 2016) as well as NFκB activation studies (Trask, 2004). HeLa cells [CCL-2, American Type Culture Collection (ATCC), Manassas, VA, USA; provided by Christian Blenn, Institute of Veterinary Pharmacology and Toxicology, University of Zurich, Zurich, Switzerland] were cultured at 37°C, 5% CO_2_ in growth medium. Growth medium consisted of Minimal Essential Medium (MEM) with Earle's salts, 25 mM HEPES, without L-Glutamine (GIBCO, Thermo Fisher Scientific, Waltham, MA, USA) supplemented with 10% fetal calf serum (FCS, BioConcept, Allschwil, Switzerland), 4 mM GlutaMAX-I (200 mM, GIBCO), and 1% MEM Non-Essential Amino Acids (100x, GIBCO).

For immunofluorescence (IF) microscopy, cells were seeded at 1 × 10^5^ cells/well in 24-well plates [Techno Plastic Products AG (TPP), Trasadingen, Switzerland] in 1 mL/well growth medium on 13 mm diameter glass coverslips (Sterilin Limited; Thermo Fisher Scientific, Cambridge, UK). For NFκB activation assay, interleukin-6 enzyme-linked immunosorbent assay (IL-6 ELISA) and associated Western blots, cells were seeded at 3 × 10^6^ cells/dish in 100 mm dishes (TPP) in 10 mL/dish growth medium. For 8 M urea lysis and associated Western blots, cells were seeded at 3.65 × 10^5^ cells in 6-well plates (TPP) in 3 mL/well growth medium. In all culture formats, seeding density was equal to 4–5 × 10^4^ cells/cm^2^. Cells were cultured 36–48 h, until monolayers approached confluence, for subsequent use in experiments.

Vero 76 cells (African green monkey kidney epithelial cells, CRL-1587, ATCC) and Caco-2 cells (human colorectal adenocarcinoma epithelial cells, HTB-37, ATCC), used in preliminary immunofluorescence microscopy experiments, were cultured and seeded in 24-well plates as described for HeLa cells.

### Chlamydial strains

*C. pecorum* 1710S, a swine abortion isolate (Kaltenboeck and Storz, 1992), and *Chlamydia suis* S45/6, a swine intestinal isolate (Kaltenboeck et al., 1993), were kindly provided by Professor J. Storz, Baton Rouge, LA, USA. The *C. trachomatis* serovar E isolate (Holt et al., 1967) was provided by Professor R. V. Schoborg, Johnson City, TN, USA.

All chlamydiae were propagated in HeLa cells in the presence of cycloheximide (CHX). Resulting crude stocks were generated by mechanical disruption (scraping into culture medium), sonication on ice for 10 min (Branson Sonifier 250; Branson Ultrasonics, Danbury, CT, USA), clearing of cellular debris by centrifugation at 500 g, 4°C for 10 min, pelleting of *Chlamydia* from the cleared lysates at 10,000 g, 4°C for 45 min and, finally, resuspension of *Chlamydia* in SPG medium prior to storage at −80°C. SPG medium consisted of 218 mM sucrose (Sigma-Aldrich, St. Louis, MO, USA), 3.76 mM KH_2_PO_4_ (Sigma-Aldrich), 7.1 mM K_2_HPO_4_ (Merck Eurolab AG, Dietlikon, Switzerland), and 5 mM GlutaMAX-100 (GIBCO). Aliquots of stocks of each *Chlamydia* species were titrated by serial dilution in HeLa cells to determine specific inclusion-forming units (IFU)/mL of each species in HeLa cells, and freshly thawed stock aliquots were used in all experiments.

To generate heat treated/inactivated *C. pecorum* stocks, crude stock was boiled for 20 min (Kol et al., 1998) in a boiling water bath or heated at 75°C for 30 min (Datta et al., 2014) in a heat block. Red fluorescent 2 μm latex particles (FluoSphere, Invitrogen) were used as a source of non-infectious particles in some experiments.

### Mycoplasma testing

Cell culture supernatants and *Chlamydia*-infected HeLa supernatants, both cultured 24–48 h with no antibiotics, were confirmed free of *Mycoplasma* contamination using the Venor GeM OneStep Mycoplasma Detection Kit for Conventional PCR (Minerva Biolabs GmbH, Berlin, Germany) per manufacturer's instructions.

### Experimental design, host cell infection, and exposure to cycloheximide, penicillin, or tumor necrosis factor alpha (see Figure 1)

Cells, cultured as described, were infected with *Chlamydia* stocks at the indicated multiplicity of infection (MOI), defined as the IFU per cell, as determined by titration in HeLa cells. In experiments comparing the effects of infection with multiple chlamydial species, equivalent IFU per cell were used for all species. Chlamydial stock was added directly to the growth medium and plates or dishes were centrifuged at 1,000 g, 25°C for 1 h as previously described (Borel et al., 2010). In some preliminary experiments, as noted in the text, centrifugation was omitted. After infection, plates or dishes were returned to 37°C, 5% CO_2_ without medium change, for 2 h of continued incubation, or medium was aspirated and replaced with fresh growth medium and plates or dishes were returned to 37°C, 5% CO_2_, for 24 h of incubation. Mock stock, generated from uninfected HeLa monolayers via the method described for stock generation, was used for mock-infected control groups. Samples (fixed cell monolayers, cell lysates, or culture medium) were collected at 1, 2, 3, or 24 hpi, depending on the experiment. In selected experiments, the effect of centrifugation was evaluated and non-centrifuged controls were kept at room temperature, covered (dark), for 1 h during the parallel centrifugation.

In uninfected host cells exposed to tumor necrosis factor alpha (TNFa) as a control for nuclear factor kappa B nuclear (NFκB) translocation/activation, human recombinant TNFa (Sigma-Aldrich) was reconstituted in filter-sterilized phosphate buffered saline (PBS, Invitrogen, Carlsbad, CA, USA) to a stock concentration of 10 μg/ml and was added to the existing growth medium at a final concentration of 20, 50, or 100 ng/ml. Subsequently, the cells were incubated at 37°C, 5% CO_2_ for 45, 60, 90, 120, or 180 min prior to sample collection, depending on the experiment. Sterile PBS alone served as the control. In some preliminary experiments, as noted in the text, cells were exposed to chlamydiae without centrifugation and compared to cells similarly exposed to TNFa.

In experimental groups exposed to cycloheximide (Sigma-Aldrich), 100 μg/ml CHX, dissolved in deionized water and filter sterilized, was added to the existing growth medium, at a final concentration of 1 or 5 μg/ml, 2 h prior to infection or TNFa exposure. Sterile water alone was added to CHX non-exposed groups. In the case of groups incubated for 24 hpi prior to sample collection, CHX exposure, or water exposure, was maintained in the fresh growth medium.

In experimental groups exposed to penicillin, penicillin G sodium salt (Sigma-Aldrich) was dissolved in deionized water to a stock concentration of 20,000 units (U)/mL, filter-sterilized, and stored at −20°C. Aliquots of this stock were thawed and further diluted in sterile water to a working concentration of 100 U/mL immediately before use. To achieve the final 1 U/ml penicillin G concentration used in all experiments, the 100 U/ml working solution was diluted in growth medium of penicillin-exposed samples and added to uninfected or *Chlamydia*-infected HeLa cells immediately prior to further incubation. Sterile water alone was similarly added to growth medium to generate control (mock penicillin-exposed) samples.

### Immunofluorescence microscopy and semi-quantitative analysis

Host cells were fixed with 4% formaldehyde for 1 h and rinsed with PBS prior to immune labeling. Reagents described herein for IF staining were dissolved in PBS and used at room temperature. Prior to primary antibody incubation, cells were exposed to 100 mM glycine for 5 min, followed by 0.1% Triton X-100 for 1 min, and cells were blocked with 0.1% Triton X-100, 1% bovine serum albumin (BSA; Sigma-Aldrich) for 30 min. Primary and secondary antibodies were diluted in 0.1% Triton X-100, 1% BSA.

For determination of NFκB localization, the primary antibody, 1:250 diluted rabbit anti-NFκB p65 (C-20, sc-372; Santa Cruz Biotechnology, Santa Cruz, CA, USA), was incubated on cells for 1 h, followed by washing 3 times with PBS, and 45 min incubation with one of two secondary antibodies, depending on the experiment: 1:500 diluted Alexa Fluor 488-conjugated goat anti-rabbit or 1:1,000 diluted Alexa Fluor 594-conjugated goat anti-rabbit (Molecular Probes, Eugene, OR, USA). Host and chlamydial DNA were labeled with 1 μg/mL 4′,6-diamidino-2′-phenylindole dihydrochloride (DAPI, Molecular Probes) included with the diluted secondary antibody. After secondary antibody incubation, cells were washed 3 times with PBS and coverslips were mounted on glass slides with FluoreGuard Mounting (Hard Set, ScyTek Laboratories Inc., Logan, UT, USA) and stored covered/dark.

Coverslips were evaluated using a Leica DMLB fluorescence microscope (Leica Microsystems, Wetzlar, Germany) under oil immersion at 1,000x magnification with a 1,006 objective (PL FLUOTAR 100x/1.30, OIL, ‘/0.17/D, Leica Microsystems) and a 106 ocular objective (Leica L-Plan 10x/ 25 M, Leica Microsystems). Microscopic images were captured using BonTec software (BonTec, Bonn, Germany) and a UI-2250SE-C-HQ camera (uEye, IDS Imaging Development Systems GmbH, Obersulm, Germany). For semi-quantitative analysis of NFκB nuclear translocation, twenty 1,000x magnification images per coverslip were randomly selected by closing transmission to the camera and oculars, moving the stage within the area of the coverslip, opening transmission to the camera, and capturing the resulting image. The five most centrally located cells in each image were scored (100 cells total, per coverslip) for nuclear translocation as follows: a “negative” (−) nuclear translocation score was given to cells with NFκB labeling primarily in the cytoplasm (little to no co-localization of NFκB with the host cell nucleus), a “positive” (+) nuclear translocation score was given to cells with NFκB labeling primarily in the nucleus (little to no co-localization of NFκB with the host cell cytoplasm), and a score of “intermediate” (=) nuclear translocation was given to cells with simultaneous NFκB labeling in both the cytoplasm and nucleus (see Supplemental Figure 2). Results are presented as percent of cells per coverslip scoring positive, negative, or intermediate NFκB nuclear translocation.

In the case of simultaneous double IF staining of chlamydiae and NFκB, 1:200 diluted primary *Chlamydiaceae* family-specific mouse monoclonal antibody directed against the chlamydial lipopolysaccharide (LPS, Clone ACI-P; Progen, Heidelberg, Germany) was diluted together with the 1:250 diluted primary rabbit anti-NFκB p65 as a single primary antibody solution, and 1:1,000 diluted Alexa Fluor 594-conjugated secondary goat anti-mouse antibody and 1:500 diluted Alexa Fluor 488-conjugated secondary goat anti-rabbit antibody, plus 1:1,000 diluted DAPI, were included in a single secondary antibody solution. Analysis and imaging were as described for NFκB single immunofluorescence staining.

Where host cell nuclei and chlamydial inclusions were enumerated, twenty 1,000x magnification fields per coverslip (at least 360 cells) were counted and an inclusion per nucleus value was generated for each field. Results are presented as mean nuclei per field or mean inclusions per field (*n* = 20, ±standard deviation).

### Host cell lysis and growth medium collection

Whole cell, cytoplasmic and nuclear extracts for NFκB activation detection and Western blotting were generated using reagents from the TransAM Nuclear Extract Kit and Lysis Buffer AM2 from the TransAM NFκB p65 Transcription Factor Assay Kit (substituted for Nuclear Extract Kit Lysis Buffer AM1, as recommended by the manufacturer for subsequent downstream use in the NFκB p65 Transcription Factor Assay Kit) per manufacturer's instructions (Active Motif, Inc., Carlsbad, CA, USA).

For cell collection, all collection and storage tubes and cell scrapers used were thoroughly pre-chilled at −20°C prior to use and kept on ice during collection and processing. Centrifuges, including buckets and inserts, were pre-cooled to 4°C and all reagents were kept on ice. The 100 mm dishes were placed on ice and growth medium was removed and held on ice for further processing as described below. Cells were washed once with 5 ml PBS supplemented with Phosphatase Inhibitor Cocktail (PBS/PI), scraped into 3 mL PBS/PI and centrifuged at 200 g, 4°C for 5 min to pellet cells. Supernatant PBDS/PI was discarded and cell pellets were kept on ice for immediate processing to whole cell or cytoplasmic/nuclear extracts, depending on experiment.

For whole cell extract generation, cell pellets were immediately thoroughly resuspended, by pipetting, in 300 μl/pellet Complete Lysis Buffer [1% Protease Inhibitor Cocktail (4-benzenesulfonyl fluoride hydrochloride, Aprotinin, Bestatin, Proteinase Inhibitor E 64, Leupeptin and Pepstatin A) and 5 mM Dithiothreitol (DTT) final concentrations in TransAM NFκB p65 Transcription Factor Assay Kit Lysis Buffer AM2]. Suspended pellets were incubated on ice, vortexed for 5 s every 10 min for 30 min (i.e., 3 times), and finally vortexed for 30 additional seconds before centrifugation at 14,000 g, 4°C for 20 min. The whole cell fractions, i.e., the supernatants, were immediately transferred, in aliquots, to chilled storage tubes and frozen/stored at −80°C.

For cytoplasmic/nuclear extract generation, cell pellets were immediately gently resuspended, by pipetting, in 500 μl/pellet 1X Hypotonic Buffer and incubated on ice 15 min, at which time 25 μl/tube of Detergent was added and samples were vortexed for 10 s prior to being centrifuged at 14,000 g, 4°C for 30 s to pellet host cell nuclei. The cytoplasmic fractions, i.e., the supernatants, were immediately transferred, in aliquots, to chilled storage tubes and frozen/stored at −80°C. The nuclear pellet was kept on ice for immediate further processing.

For nuclear extract generation, nuclear pellets were immediately thoroughly resuspended, by pipetting, in 25 μl/pellet Complete Lysis Buffer plus 2.5 μl/pellet Detergent, pellets were vortexed 10 s and incubated on ice, vortexed for 5 s every 10 min for 30 min (i.e., 3 times), and finally vortexed for 30 additional seconds before centrifugation at 14,000 g, 4°C for 10 min. The nuclear fractions, i.e., the supernatants, were immediately transferred, in aliquots, to chilled storage tubes and frozen/stored at −80°C.

For 8M urea lysis, previously reported to strongly limit the activity of chlamydial proteasome-like activity factor (CPAF), freshly prepared 8M urea (Sigma-Aldrich) was supplemented with 325 U/mL final concentration of Benzonase Nuclease (Sigma-Aldrich) and used to lyse 6-well plates of HeLa essentially as described (Chen et al., 2012). Briefly, the 6-well plates were placed on ice, growth medium was aspirated from wells, cells were washed with 3 mL/well ice-cold sterile PBS (1X Dulbecco's PBS, without calcium or magnesium; GIBCO, Invitrogen), PBS was aspirated from wells, 1 mL/well 8M urea with 325 U/mL Benzonase Nuclease was added per well and incubated on ice 10 min, and finally extracts were immediately transferred to chilled storage tubes and stored at 4°C. Urea whole cell extracts were evaluated by Western blot only.

Aliquots of all extracts were evaluated by BCA Protein Assay Kit (Pierce Biotechnology, Rockford, IL, USA), per manufacturer's instructions, using an Epoch 2 Microplate Spectrophotometer (BioTek Instruments, Inc., Winooski, VT, USA) and Gen5 software (BioTek Instruments, Inc.).

For growth medium processing and storage (for IL-6 ELISA), collected growth medium (as described above) was kept on ice prior to sequential syringe filtration, first with 0.22 μm filters (TPP) and then with 0.1 μm filters (GE Healthcare, Little Chalfont, UK), to remove chlamydial RBs and EBs (Buckner et al., 2013; Marti et al., 2014) prior to being frozen/stored at −80°C.

### Nuclear factor kappa B activation assay

The TransAM NFκB p65 Transcription Factor Assay Kit (Active Motif, Inc.) was used to evaluate NFκB p65 activation in whole cell, cytoplasmic and nuclear extracts collected at 2 or 24 hpi. The kit contains an ELISA-style 96-well plate coated with the NFκB consensus site (5′-GGGACTTTCC-3′), to which active NFκB specifically binds. An NFκB p65 primary antibody is used for detection, and binds a p65 epitope only accessible when NFκB is activated and bound to the target sequence.

Stored extracts were thawed immediately prior to use, kept on ice, and the assay was performed per manufacturer's instructions. Briefly, 15 μg of extract per well was diluted in 20 μl Complete Binding Buffer (10 ng/mL Herring sperm DNA and 2 mM DTT final concentrations in Binding Buffer AM3) and this was added to wells containing 30 μl Complete Binding Buffer per well (i.e., added to the wells in 50 μl total volume per well). After incubation at room temperature for 1 h, well contents were decanted and wells were washed. Wells were then sequentially incubated with primary and secondary antibodies (horseradish peroxidase-conjugated secondary) and Developing Solution. Absorbance was read at 450 nm, with blank subtraction, on an Epoch 2 Microplate Spectrophotometer (BioTek Instruments, Inc.). Samples were assayed in duplicate. The results are presented as blank-subtracted 450 nm absorbance values set relative to the mock value.

### Interleukin-6 enzyme-linked immunosorbant assay (ELISA)

The Qiagen Human IL6 Single Analyte ELISArray Kit (Qiagen, Hilden, Germany), a standard sandwich ELISA assay, was used to determine the IL-6 concentration in culture supernatant (growth medium) collected at 2 or 24 hpi. Stored medium was thawed immediately prior to use, and the assay was performed per manufacturer's instructions. Recommended dilutions of the provided Antigen Standard diluted in Sample Dilution Buffer 1 (final concentration of 1% bovine serum albumin in Sample Dilution Buffer Stock) were used for standard curve generation, as indicated by the manufacturer for assay of cell culture supernatant samples. Plates were read on an Epoch 2 Microplate Spectrophotometer (BioTek Instruments, Inc.) and analyzed with Gen5 software (BioTek Instruments, Inc.). Samples were assayed in duplicate. The data were analyzed as IL-6 secretion relative to uninfected control samples (uninfected samples exposed to no additives and not subjected to centrifugation) and results are presented as IL-6 secretion relative to mock-infected samples.

### Western blot analysis

Stored TransAm Kit-generated whole cell, cytoplasmic or nuclear extracts and Urea whole cell extracts were moved to ice immediately before use. Eight micrograms of each extract was diluted in 4X Laemmli Sample Buffer (Bio-Rad, Hercules, CA, USA) supplemented with DTT (Sigma-Aldrich) to 50 mM DTT final concentration, incubated at 95°C for 5 min for protein denaturation, centrifuged 30 s at 14,000 g, and loaded into 4–20% Mini-PROTEAN TGX Precast Gels (Bio-Rad). Precision Plus Protein Dual Color Standards (10–250 kDa; Bio-Rad) or PageRuler Plus Prestained Protein Ladder (10–250 kDa; Thermo Fisher Scientific) were included on each gel for molecular weight estimation. Proteins were separated by sodium dodecyl sulfate-polyacrylamide gel electrophoresis and transferred to nitrocellulose membranes, which were dried overnight.

Membranes were stained with REVERT Total Protein Stain (LI-COR Biosciences, Lincoln, NE, USA), per manufacturer's instructions, imaged with an Odyssey CLx Infrared Imaging System and analyzed for total protein signal with Image Studio Software (both, LI-COR Biosciences) with User-Defined background method, as recommended by the manufacturer. Membranes were incubated with REVERT Reversal solution (LI-COR Biosciences) for 5–10 min and rinsed briefly with water and processed immediately for immunodetection, as follows.

Membranes were blocked with 1X TBS plus 5% skim milk at room temperature for 1 h. Antibodies were diluted in 1X TBS plus 0.2% Tween-20 (Sigma-Aldrich) and 5% skim milk. NFκB p65 was probed with 1:100 diluted rabbit anti-NFκB p65 primary antibody (same antibody as described in Immunofluorescence Microscopy and Semi-Quantitative Analysis Section) overnight at 4°C and 1:20,000 diluted IRDye 800CW Donkey anti-Rabbit IgG secondary antibody (LI-COR Biosciences) for 1 h at room temperature. Membranes were washed 4 times, for 5 min, with 1X TBS plus 0.1% Tween-20 after primary and secondary antibody incubations and washed once, for 5 min, with 1X TBS prior to imaging on an Odyssey CLx Infrared Imaging System and analyzed for NFκB band protein signal with Image Studio Software (both, LI-COR Biosciences) with Average background method, as recommended by the manufacturer. Single, distinct bands of ~65 kDa were labeled with the NFκB p65 antibody, as expected.

Quantitative analysis for each Western blot was performed by normalizing the NFκB signal to the membrane total protein signal for each lane as per manufacturer's recommendation. Quantitative results are presented as normalized NFκB signal values.

### Statistical analysis

Statistical analyses were performed using Microsoft Excel. Significance of the difference of means was determined by two-tailed, unpaired *t*-test and *p* < 0.05 were considered significant. *p*-values were confirmed using the GraphPad QuickCalcs Web site: http://www.graphpad.com/quickcalcs/ttest1/ (accessed 2016). Unless stated otherwise, results are displayed as means, ±standard deviation, of the results from the stated number of independent experiments.

## Results

### Preliminary experiments and controls

Immunofluorescence (IF) microscopy was used to evaluate the ability of porcine chlamydiae species *C. suis* S45/6 strain (Kaltenboeck et al., 1993) and *C. pecorum* 1710S strain and the human pathogen *C. trachomatis* serovar E (Holt et al., 1967) to induce nuclear translocation of the inflammatory transcription factor NFκB p65 subunit. First, we evaluated HeLa cells, as well as a human colon carcinoma cell line (Caco-2) and a monkey kidney epithelial cell line (Vero-76), for response to the known NFκB activator TNFa (Lowenthal et al., 1989). Exposure to TNFa, added directly to the culture medium at doses ranging from 20 to 100 ng/mL, induced the expected HeLa p65 nuclear translocation at 45 min post exposure. Specifically, in mock-exposed cells, NFκB labeling was largely absent from the nucleus and visible in the cytoplasm, while in TNFa-exposed cells NFκB labeling was observed in both the nucleus and cytoplasm. This effect was similar at 60 min post exposure, but returned to levels equivalent to mock-exposed cells by 90 min post exposure (Supplemental Figure 1A). Vero and Caco cells were unresponsive to exposure to TNFa at these concentrations, during these times (Supplemental Figure 1B), showing no detectable NFκB nuclear translocation upon exposure. Thus, HeLa cells were used in all subsequent assays for this study and NFκB labeling in these cells was confirmed to be specific (Supplemental Figure 1C). For initial evaluation of the potential effect of chlamydiae on HeLa NFκB nuclear translocation, we exposed HeLa to *C. pecorum* in the same manner as described for TNFa exposure, by adding chlamydial stock, at a MOI of 5 inclusion forming units (IFU) per cell, directly to the culture medium and incubating for 45 or 60 min. *C. pecorum* infection failed to induce NFκB nuclear translocation under these conditions (Supplemental Figure 1C).

### *Chlamydia pecorum* induces nuclear factor kappa B (NFκB) nuclear translocation and activation early after infection

**Figure 1:**
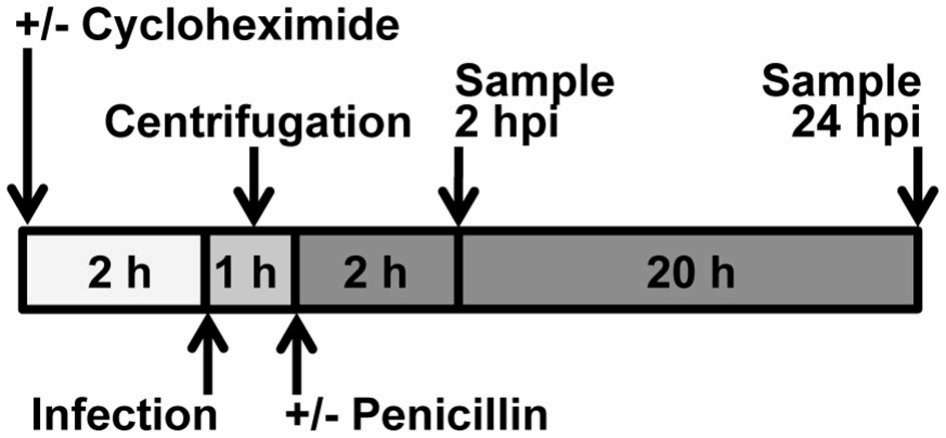
**Experimental Design**. Cycloheximide was added to culture medium 2 h before infection. *Chlamydia* was added to culture medium immediately prior to centrifugation or incubation. When centrifugation was omitted, incubation was continued immediately after addition of *Chlamydia*. When centrifugation was a variable, non-centrifuged samples were placed adjacent to the centrifuge, covered, during the centrifugation. For 2 hpi experiments, medium was not changed; for 24 hpi experiments, medium was changed immediately after centrifugation. Penicillin was added to culture medium after centrifugation. Upon medium change, cycloheximide level was maintained.

Pre-exposure to 1 or 5 μg/mL of the Eukaryotic protein synthesis inhibitor cycloheximide (CHX) for 2 h markedly increased the degree of NFκB nuclear translocation upon TNFa exposure, with NFκB labeling observed primarily in the nucleus and largely absent from the cytoplasm (Supplemental Figure 2A), representing a more robust translocation than that observed for TNFa exposure in the absence of cycloheximide (Supplemental Figure 1A). Initial experiments with 2 h CHX pre-exposure, and a time range of 1, 2, or 3 h post-exposure, confirmed NFκB nuclear translocation occurred by 1 h but was more pronounced at 2 and 3 h (not shown). Thus, 2 hpi was selected as the time for evaluation of NFκB nuclear translocation early after chlamydial infection (see Experimental Design, Figure 1).

**Figure 2:**
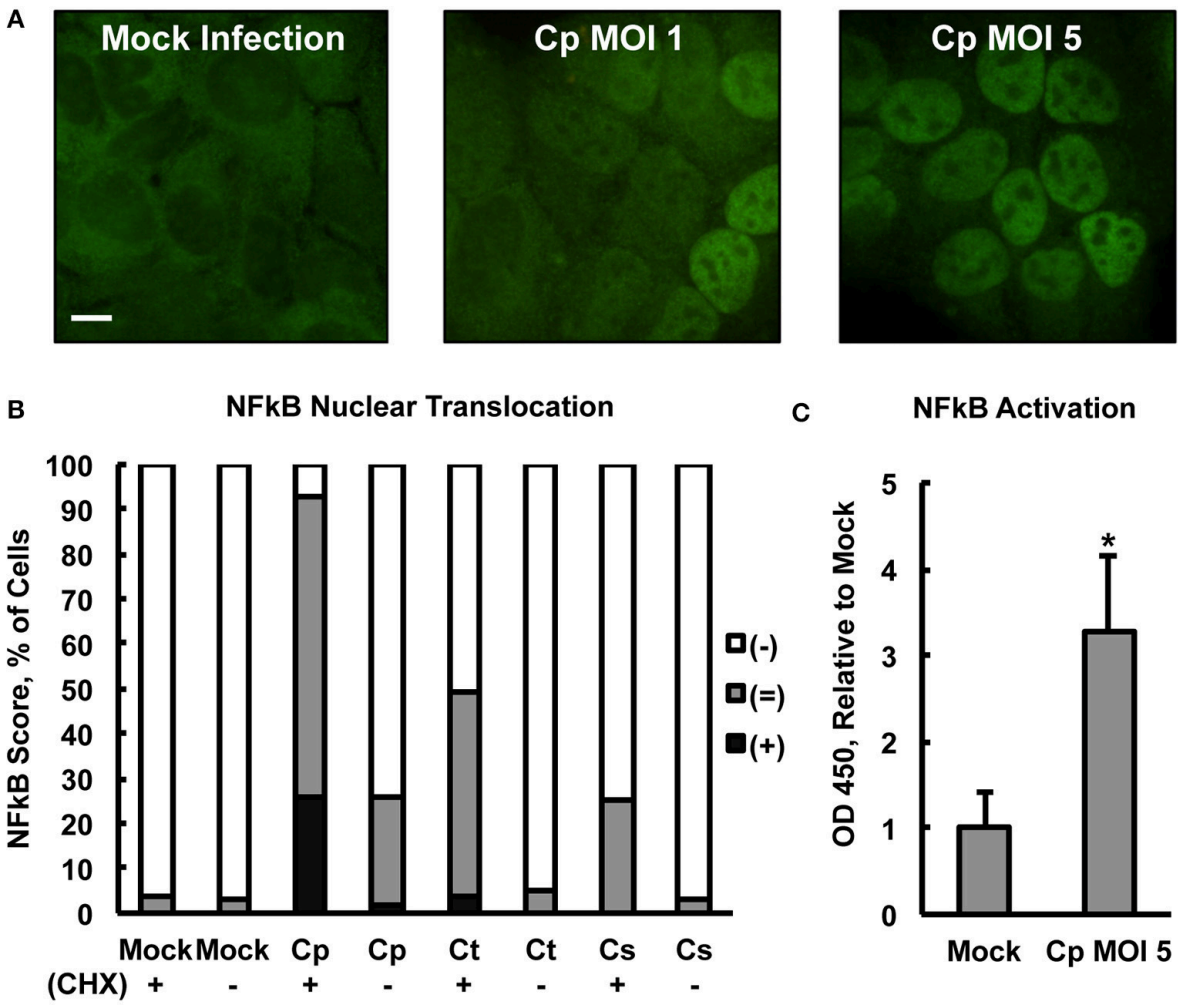
***Chlamydia pecorum* Induces Nuclear Factor Kappa B (NFκB) nuclear translocation and activation early after infection**. HeLa cells were pre-exposed (+), or not (−), to 1 **(C)** or 5 **(A,B)** μg/mL cycloheximide (CHX) for 2 h, infected using centrifugation with *C. pecorum* (Cp), *C. trachomatis* (Ct), or *C. suis* (Cs) at a multiplicity of infection (MOI) of 1 or 5 and incubated for 2 h. **(A)** Immunofluorescence (IF) microscopic analysis, in which NFκB p65 was labeled (green), showed *C. pecorum*-dependent NFκB nuclear translocation (CHX+). **(B)** Semi-quantitative analysis of NFκB nuclear translocation assayed by IF microscopy, wherein 100 cells per group were scored positive (+), intermediate (=), or negative (−) for NFκB nuclear translocation (see Section Material and Methods), showed that *C. pecorum* induced the most robust NFκB nuclear translocation of the chlamydial species (MOI 5) evaluated. CHX pre-exposure potentiated *Chlamydia*-dependent NFκB nuclear translocation. **(C)** Quantitative analysis of NFκB activation (CHX +) was measured by an Enzyme-linked Immunosorbent Assay (ELISA)-style assay wherein NFκB binding the target DNA consensus sequence, immobilized in a plate, was detected by immune labeling of the bound, active NFκB p65 subunit. *C. pecorum* infection significantly increased NFκB activation compared to the mock-infected control (*n* = 3, mean ± standard deviation, ^*^p < 0.05).

Centrifugation may be used to increase chlamydial uptake and inclusion formation (Allan and Pearce, 1979; Moulder, 1991). Therefore, centrifugation-assisted infection and CHX pre-exposure were used simultaneously in an effort to maximize the potential measurable effect of *Chlamydia* on NFκB nuclear translocation. Upon CHX pre-exposure of HeLa with subsequent centrifugation-assisted infection (MOI of 1 or 5), *C. pecorum* induced NFκB nuclear translocation at 2 hpi (Figure 2A) in a dose-dependent manner. Semi-quantitative IF microscopy analysis of *C. pecorum, C. suis*, and *C. trachomatis* infection under these conditions (Figure 2B) showed that *C. pecorum* infection induced substantial NFκB nuclear translocation, with 67% of cells scoring (=) and 26% of cells scoring (+), compared to the mock-infected control in which only 4% of cells scored (=), and no cells scored (+). *C. trachomatis* and *C. suis* induced less of an effect, with 45%(=)/4%(+) and 25%(=), respectively. Omission of cycloheximide pre-exposure reduced the degree of NFκB nuclear translocation associated with all three chlamydial species evaluated, with only *C. pecorum* [2%(+)/24%(=)] showing nuclear translocation notably higher than the mock-infected control. Omission of centrifugation from the infection protocol had a similar effect on all three chlamydial species, reducing NFκB nuclear translocation to levels similar to that of the mock-infected control (Supplemental Figure 3A). To determine if the observed potentiation of *Chlamydia*-dependent NFκB nuclear translocation by centrifugation could be attributed specifically to chlamydial factors, infection, with CHX pre-exposure and centrifugation, was carried out using heat treated/inactivated *C. pecorum* stock (MOI 5) or 2 μm red fluorescent latex particles. Neither heat treated/inactivated stocks, nor either concentration of latex particles, increased NFκB nuclear translocation relative to mock infection (Supplemental Figure 3B), indicating that active chlamydiae are required to induce translocation at 2 hpi and inactive/non-infective particles alone are insufficient to do so. IF microscopy confirmed that centrifugation potentiated latex particle association with cells, and multiple particles were associated with all cells observed upon centrifugation (not shown).

An Enzyme-linked Immunosorbent Assay (ELISA)-style assay of HeLa lysates (lysed per manufacturer's instructions specifically to maintain NFκB activity, see Section Material and Methods) was used to confirm that early *C. pecorum*-dependent NFκB nuclear translocation was associated with the expected increase in DNA consensus sequence binding activity, specifically of the p65 subunit. Evaluated as an assay control, exposure to 20 ng/mL TNFa, after 2 h CHX pre-exposure, induced the expected NFκB activation at 2 h post exposure (Supplemental Figure 4A). This TNFa-induced activation was most apparent in the nucleus, where it reached >4 times the mock-exposed value, as opposed to the cytoplasm, where it reached <2 times the mock-exposed value, as expected and in agreement with the IF microscopy findings. Assay of TNFa-induced NFκB activation in whole cell lysate, >5 times the mock-exposed value, reflected overall NFκB activation as expected, and whole cell lysates were selected to further evaluate the effect of *C. pecorum* on NFκB activation, with the goal of minimizing lysate processing time. In agreement with the IF microscopy findings, *C. pecorum* infection induced whole cell NFκB activation to >3 times the mock-infected value (Figure 2C). Similar to the TNFa control, *C. pecorum*-infected cytoplasmic and nuclear lysates showed the expected relatively low (<1.5 times the mock-infected value) and high (>4 times the mock-infected value) levels of NFκB activation, respectively (Supplemental Figure 4B), in agreement with IF microscopy findings. Also in agreement with the IF microscopy findings, the ability of *C. pecorum* to induce NFκB activation was found to be dose dependent (Supplemental Figure 4C), while *C. suis* induced a much less robust effect (Supplemental Figure 4D). Taken together, these data indicate that *C. pecorum* induces NFκB nuclear translocation and activation in HeLa cells early after infection.

### *Chlamydia pecorum*-induced nuclear factor kappa B activation and interleukin-6 secretion are detectable at 24 h post infection, effects not abolished by penicillin-induced chlamydial stress

**Figure 3:**
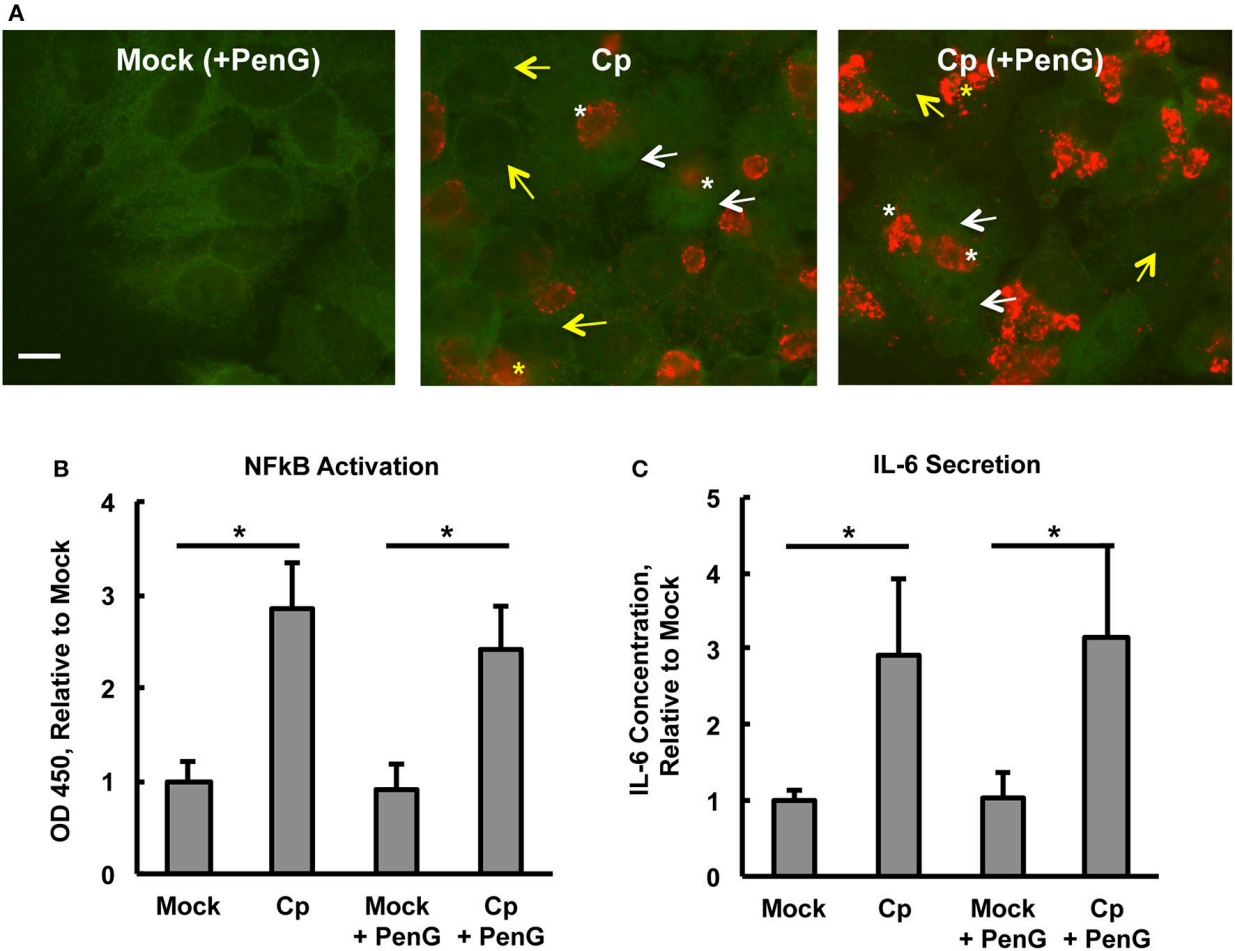
***Chlamydia pecorum*-Induced Nuclear Factor Kappa B (NFκB) Activation and Interleukin-6 Secretion are Detectable 24 hpi, Effects not Abolished by Penicillin-Induced Chlamydial Stress**. HeLa cells were pre-exposed **(A)** or not **(B,C)** to 1 μg/mL cycloheximide (CHX) for 2 h, infected using centrifugation with *C. pecorum* (Cp) at a multiplicity of infection (MOI) of 1 and incubated until 24 h post infection (hpi) with or without exposure to 1 unit/mL penicillin G (PenG) in the incubation medium. **(A)** Representative IF micrographs show NFκB p65 (green) and *C. pecorum* LPS (red) at 1,000x magnification. White and yellow arrows indicate the nuclei of cells with and without NFκB nuclear translocation, respectively. White and yellow asterisks indicate chlamydial inclusions in cells with and without NFκB nuclear translocation, respectively. Scale bar = 10 μm. **(B)** NFκB activation, specifically of subunit p65, was assayed by an enzyme-linked immunosorbent assay (ELISA)-style assay of whole cell lysates and showed that *C. pecorum* significantly induced NFκB activation, regardless of PenG-induced chlamydial stress (*n* = 3, mean ± standard deviation, ^*^p < 0.05). **(C)** Interleukin-6 (IL-6) secretion was assayed by ELISA evaluation of cell culture medium and showed that *C. pecorum* significantly induced IL-6 secretion, regardless of PenG-induced chlamydial stress (*n* = 3, mean ± standard deviation, ^*^p < 0.05).

To determine if the observed *C. pecorum*-dependent early NFκB p65 activation of HeLa cells was sustained until a later chlamydial developmental stage, and to determine if penicillin G (PenG)-induced chlamydial stress could influence potential activation, we selected 24 hpi for evaluation of continued NFκB activation. At this time, inclusions were easily detectable and chlamydial replication was underway (Supplemental Figures 5A,B). Initial evaluation of the effect of CHX pre-exposure/exposure (CHX maintained throughout the experiment) and PenG post-infection exposure on HeLa and *C. pecorum* growth/development at 24 hpi showed that (i) host cell numbers were reduced upon CHX pre-exposure, as expected, since CHX was maintained throughout the experiment reducing eukaryotic protein synthesis and thus cellular division, (ii) inclusions numbers were roughly similar between CHX-pre-exposed and unexposed groups (~12.5–13.5 inclusions per 1,000X field) despite the difference in host cell numbers, (iii) PenG had no effect on host cell numbers or inclusion numbers, and (iv) *C. pecorum* infection did not cause host cell loss relative to mock infection at 24 hpi. ABs, indicative of chlamydial stress, were exclusively observed in PenG-exposed groups, regardless of CHX exposure. Thus, PenG induced the expected chlamydial stress, as characterized by AB formation without the prevention of inclusion formation (Leonard et al., 2016). NFκB nuclear translocation was increased in *C. pecorum*-infected HeLa, relative to mock-infected controls, in both PenG-exposed and unexposed groups (Figure 3A). Additionally, in both *C. pecorum*-infected groups, NFκB nuclear translocation was not restricted to infected cells, but was observed in both infected and uninfected cells (Figure 3A). Because inclusions partially obscured determination of NFκB cytoplasmic localization, making it difficult to determine (+) vs. (=) NFκB nuclear translocation, semi-quantitative analysis was not undertaken for 24 hpi groups.

ELISA measurement of NFκB activation was carried out on whole cell lysates to confirm that *C. pecorum*-dependent NFκB nuclear translocation at 24 hpi was associated with the expected increase of NFκB activation. Infection with the relatively low MOI of 1 was used in order to limit potential infection-induced cell lysis, which could trigger danger signaling due to extracellular detection of normally cytoplasmic components (Broggi and Granucci, 2014). Such signaling has been shown to strongly impact *C. pecorum* development in HeLa (Leonard et al., 2015). An initial evaluation of the potential requirement for CHX pre-exposure/exposure for *C. pecorum*-induced NFκB activation at 24 hpi was made. While NFκB activation at this later time was strongly potentiated by CHX, as observed for the 2 hpi time point, marked activation occurred even in the absence of CHX (Supplemental Figure 6A). It is notable that CHX pre-exposure/exposure was associated with an increase in the mock infection NFκB activation to >2 times the corresponding value for the CHX unexposed mock-infected group. While *C. pecorum* infection in the presence of CHX resulted in an increase of ~5 times the CHX pre-exposure/exposure mock-infected group value. Further evaluation was carried out in the absence of CHX to allow subsequent evaluation of the induction of cytokine secretion in the absence of translational inhibition. *C. pecorum* infection (centrifugation-assisted), in both the presence or absence of PenG exposure, similarly increased NFκB activation to ~3 times the corresponding mock-infected value at 24 hpi (Figure 3B). Analysis of 24 hpi *C. pecorum*-infected HeLa cytoplasmic and nuclear lysates showed ~5.5 times the nuclear NFκB activation of the mock-infected group, and ~3 times the cytoplasmic activation of the mock-infected group (Supplemental Figure 6B).

IL-6 is an inflammatory cytokine, inducible by multiple factors, including but not limited to NFκB activation, and is secreted by epithelial cells in response to both naturally occurring *C. trachomatis* infection (Refaat et al., 2016) and to *in vitro C. trachomatis* infection of HeLa cells (Marti et al., 2014). Evaluation of HeLa culture supernatants by ELISA (Figure 3C) showed that IL-6 secretion was induced by *C. pecorum* infection (in the absence of CHX) to an average of 540–550 pg/mL, ~3 times the level of mock-infected HeLa. Neither mock-infected nor *C. pecorum* infected values were influenced by PenG exposure. Similar to the NFκB activation results at 24 hpi, CHX pre-exposure/exposure was associated with an increase in IL-6 secretion to ~3 times the corresponding value for the CHX unexposed mock-infected group; *C. pecorum* infection in the presence of CHX also resulted in an increase of IL-6 secretion relevant to the CHX pre-exposure/exposure mock-infected group value (Supplemental Figure 6C). These results demonstrate that exposure of HeLa to CHX does not prevent induction of subsequent IL-6 secretion, despite the limiting effect of CHX on protein synthesis and cell division. The increase in IL-6 secretion upon CHX is not unexpected, as it has been previously reported that CHX exposure is associated with super induction of primary response genes such as cytokines, including IL-6, an effect that may result from both decreased mRNA degradation and increased transcription of these genes (Herschman, 1991; Hershko et al., 2004). Collectively, these data indicate that *C. pecorum*-induced NFκB activation in HeLa is still detectable at 24 hpi, a time when IL-6 secretion can also be demonstrated; penicillin-induced chlamydial stress did not abolish either NFκB activation or IL-6 secretion.

### *Chlamydia*-infected HeLa lysates do not show decreased nuclear factor kappa B (NFκB) levels

**Figure 4:**
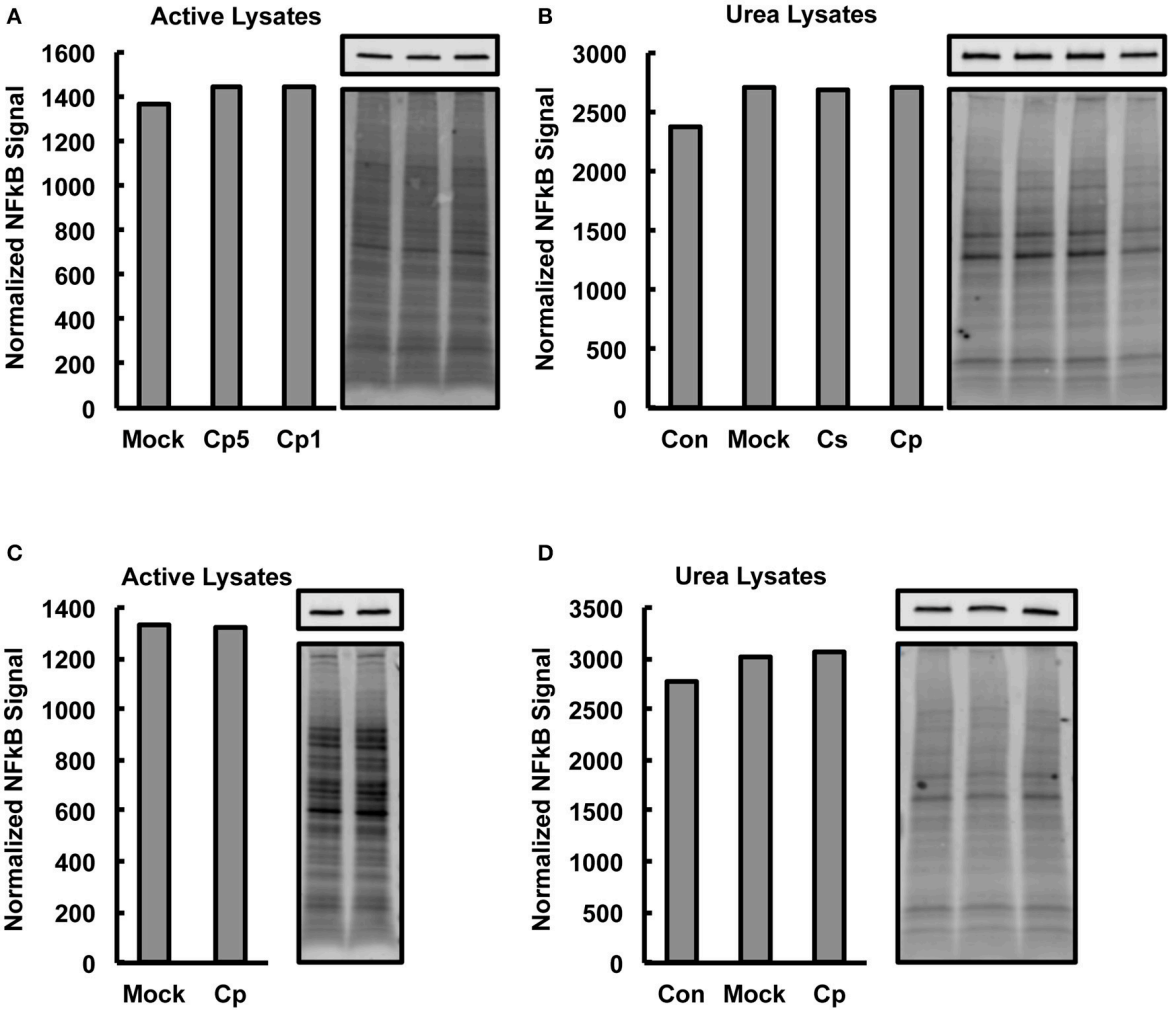
***Chlamydia*-Infected HeLa Lysates Do Not Show Decreased Nuclear Factor Kappa B (NFκB) Levels**. HeLa cells were pre-exposed to 1 μg/mL cycloheximide (CHX), or not, for 2 h, infected using centrifugation with *C. pecorum* (Cp) or *C. suis* (Cs) at a multiplicity of infection (MOI) of 1 or 5 and incubated until 2 or 24 h post infection (hpi). In addition to mock-infected controls, extra controls with no CHX added and no centrifugation (Con) were included in some experiments. Cells were lysed with a commercially available kit intended to maintain NFκB activity (active lysates) or with a urea lysis protocol reported to deactivate chlamydial protease activity factor (urea lysates). Western blotting was performed on gels loaded with 8 μg lysate protein per lane. After transfer to membranes, total protein staining (bottom image panels) was carried out on membranes prior to probing for NFκB p65 (top image panels). Graphs show NFκB signals normalized to total protein signals and bars of graphs correspond, in order, to the lanes of adjacent images. **(A,B)** 2 hpi, CHX pre-exposure, MOI 1 (Cp1 or not noted) unless indicated as MOI 5 (Cp5). **(C,D)** 24 hpi, no CHX, MOI 1. No reduction in NFκB levels was associated with chlamydial infection in any group. Active lysates data shows representative blots from two experiments and urea lysates data shows blots from single, confirmatory experiments.

The observed marked increases in NFκB p65 activation upon *C. pecorum* infection at early and late experimental time points, which would not be expected if marked degradation of NFκB p65 were occurring, suggests that significant CPAF-dependent NFκB p65 degradation was not occurring in our experimental setting. However, CPAF is known to cleave NFκB p65, has been demonstrated to be active after standard (protein denaturing) cell lysis of *C. trachomatis* infected cells and is capable of degrading some targets such as golgin-84 even when samples are kept on ice (Chen et al., 2012). Therefore, we evaluated, by Western blot analysis, −80°C stored aliquots of the non-denatured lysates generated for NFκB activity analysis for evidence of NFκB p65 degradation associated with *C. pecorum* infection of cells. We additionally analyzed *C. pecorum* infected HeLa lysates generated per a protocol shown to deactivate CPAF (Chen et al., 2012; Johnson et al., 2015), for comparison. Total protein staining was carried out on membranes prior to probing for NFκB p65, and NFκB signals were normalized to total protein signals to allow quantitative analysis of NFκB p65 (Figures 4A–D). Reduction in NFκB levels was not associated with chlamydial infection in any group, regardless of lysis protocol or time post infection of lysis, suggesting that CPAF activity against NFκB is minimal under the lysis protocol used herein for NFκB activity analysis.

## Discussion

The importance of NFκB in the inflammatory response to infection is without question. The inflammatory nature of chlamydial infection and associated disease has prompted substantial interest in the potential specific role of NFκB in chlamydial pathogenesis, especially in the context of human-infecting chlamydial species. But although chlamydial infections in animals are also common, and similarly inflammatory in nature, little evaluation of the role of NFκB in the pathogenesis of agriculturally important chlamydial species has been undertaken to date. Previous reports of chlamydial species-specific differences in NFκB-activating potential (Molestina et al., 2000; Heine et al., 2003) suggests that NFκB activation is likely not equivalent amongst the chlamydiae and may represent distinctions in pathological potential. The aim of this study was to determine if porcine chlamydial species, such as *C. pecorum* and *C. suis*, are capable of inducing NFκB activation and inflammatory cytokine secretion in the context of *in vitro* infection of epithelial cells.

*C. psittaci* and *C. muridarum*, though not well-studied, have been reported to induce NFκB activation (Heine et al., 2003; Mackern-Oberti et al., 2006; Shirey et al., 2006; Welter-Stahl et al., 2006). *C. trachomatis* and *C. pneumoniae*, however, have been well-studied amongst the chlamydiae with regards to activation of NFκB. *C. pneumoniae*, an extremely widely distributed agent of human respiratory infection, as well as an infectious agent in non-human animals, has also been implicated in other inflammatory diseases such as atherosclerosis (Leonard and Borel, 2014) and osteoporosis (Di Pietro et al., 2013). NFκB activation has been consistently reported in response to *C. pneumoniae* infection *in vitro*, over a wide range of infection times and in a wide variety of cell types, including epithelial cells (Gencay et al., 2003), monocytes (Donath et al., 2002), and especially endothelial cells, where infection is also often associated with IL-6 induction (Dechend et al., 1999; Kol et al., 1999; Visseren et al., 2002; Niessner et al., 2003). *C. trachomatis*, on the other hand, has been reported both to increase NFκB activation (Heine et al., 2003; Sellami et al., 2014) or not to do so (Molestina et al., 2000; Xiao et al., 2005), and even to degrade NFκB p65 (Lad et al., 2007; Christian et al., 2010), though this phenomenon was later found to be an artifact of *in vitro* proteolysis and not a case of true biological targeting (Chen et al., 2012). Variations in cell lines, timing post infection of NFκB assay and NFκB assay used, often varying widely from study to study, as well as the potential influence of the chlamydial protease CPAF, may all play a role in the inconsistent findings reported for *C. trachomatis*/NFκB studies.

The p65 subunit of NFκB (also known as RelA) is one of the most common of the five NFκB subunits, along with the p50 subunit, found in the NFκB signaling pathway (Hayden et al., 2006). NFκB nuclear translocation occurs upon ubiquitination and degradation of the inhibitors of NFκB (such as IkBa, inhibitor kappa B, alpha), which, until activation is initiated, maintain the localization of NFκB in the cytoplasm (Gilmore, 2006). Thus, translocation of p65 is a useful indicator of NFκB activation in general. The CPAF was recently shown to specifically degrade NFκB p65 *in vitro* during standard host cell lysis protocols, despite the fact that this cleavage does not appear to occur within intact host cells (Chen et al., 2012; Tan and Sütterlin, 2014; Johnson et al., 2015), thus IF microscopy has the advantage of providing a representative measure of NFκB activation in the absence of cell lysis. Because studies of NFκB activation by the chlamydiae must necessarily consider the potential impact of CPAF activity in assays that utilize cell lysates, steps were taken in this study to monitor lysates for potential NFκB degradation. We did not find evidence of *Chlamydia*-associated NFκB degradation, and several factors may have influenced this. First, the nature of the lysis process used for NFκB assay is not a standard protocol and is designed to maintain NFκB activity. It is conceivable that this may influence the NFκB degrading activity of CPAF in these lysates, since it is yet unknown what factors prevent CPAF-dependent NFκB degradation in intact cells (Tan and Sütterlin, 2014). Second, while *C. trachomatis*-associated NFκB degradation was shown, depending on the genotype, to begin around 20–24 hpi (Lad et al., 2007) (now known to almost surely be occurring after lysis), *C. pneumoniae* lysates evaluated in parallel did not show NFκB degradation until between 30 and 46 hpi. This suggests that species- and strain-specific differences in the timing of CPAF activity are likely, thus *C. pecorum* CPAF activity may be associated with later times post infection. And finally, though CPAF has been shown to remain active at low temperatures and to withstand conditions which inhibit other proteases (Chen et al., 2012; Tan and Sütterlin, 2014; Johnson et al., 2015), *C. pecorum* CPAF, specifically, may exhibit less proteolytic activity, at least against NFκB, at low temperatures and under certain experimental conditions. And while it is possible that undetected chlamydial-dependent NFκB degradation may have occurred during NFκB activation assays carried out this study, this would be expected to result in an underestimation of NFκB activity and would not change the basic findings of the study- which demonstrated an increase in NFκB activation attributable to *C. pecorum* infection.

That *C. trachomatis*, and the closely related *C. suis*, showed little potential to induce NFκB nuclear translocation early after infection is not unexpected, given that reports of the inability of *C. trachomatis* to induce NFκB activation have been relatively frequent. The more distantly related *C. pecorum* was associated not only with NFκB activation early after infection, but also with continued NFκB activation at 24 hpi. Notably, *C. pecorum* infection causes significant lysis of HeLa cells by 39 hpi, a time when similar infection with *C. trachomatis* has little to no lysing effect on HeLa (Leonard et al., 2015, 2016). The concomitant induction of IL-6 secretion associated with this *C. pecorum*-dependent NFκB activation cannot be directly attributed to activity of NFκB in the context of this study. However, NFκB is a known contributor to IL-6 induction and has previously been shown specifically to play a role in *Chlamydia*-dependent IL-6 induction *in vitro* (Niessner et al., 2003). The findings of this study support the existence of a relatively robust general pro-inflammatory effect of *C. pecorum* on HeLa cells, but do not specify the target(s) of NFκB transcriptional regulation in response to chlamydial infection nor clarify the requirement for NFκB activation in the observed IL-6 induction. The induction of IL-6 may alternatively, or additionally, be exerted via other modulators of IL-6 such as NF-IL6, a transcription factor important in the regulation of expression of IL-6 and other cytokines such as IL-8 and TNF (Akira et al., 1990). Analysis of the binding sites of *Chlamydia*-activated NFκB in HeLa would identify targets regulated in this model and potentially clarify the role of NFκB in the observed IL-6 induction.

The failure of heat inactivated *C. pecorum* and non-infectious latex particles to cause NFκB nuclear translocation in HeLa at 2 hpi, even with the potentiating effect of CHX and centrifugation, indicates that viable *C. pecorum* are required for the NFκB nuclear translocation demonstrated herein. Previously, heat or formalin inactivated *C. pneumoniae* were shown to induce NFκB nuclear translocation in porcine endothelial cells (Baer et al., 2003), while heat inactivated *C. pneumoniae* was incapable of activating an NFκB luciferase reporter assay in human vascular endothelial cells (Opitz et al., 2005). This suggests that the host cells, and potentially the method of NFκB activation assay, may play a role in the requirement for chlamydial viability in NFκB activation.

Penicillin-induced chlamydial stress, also termed persistence, did not significantly reduce *C. pecorum*-dependent NFκB activation, nor did it alter IL-6 secretion. This suggests (i) that either *Chlamydia*-associated factors responsible for these effects happen as a result of early events such as cell surface binding, bacterial uptake or inclusion formation, events that would occur prior to the induction of penicillin-induced chlamydial stress (associated with RB division), and/or (ii) that responsible factors remain active even during chlamydial stress. Evaluation of the requirement for chlamydial viability after chlamydial uptake may help clarify this point. Pathogen recognition receptors are potential players in the NFκB response to *C. pecorum* observed early after infection. NFκB signaling is known to occur in response to stimulation of toll-like receptors, the tumor necrosis factor receptor and the interleukin-1 receptor (Hoesel and Schmid, 2013). *Chlamydia* is recognized by toll-like receptors 2 and 4 in the female genital tract (Leonard and Borel, 2014) and can modulate both interleukin-1 beta and TNFa receptor expression in HeLa cells (Shirey and Carlin, 2006), highlighting the potential involvement of these receptors in *Chlamydia*-dependent NFκB activation.

It has been demonstrated that penicillin-induced chlamydial stress significantly increased IL-6 levels in *C. trachomatis* E-infected HeLa cells (Wasson et al., 2012). Another recent report also indicated that the NOD-2-dependent NFκB activating capacity of *C. trachomatis* L2 was significantly potentiated by ampicillin (beta lactam) exposure, again linking chlamydial stress with increased inflammatory potential (Packiam et al., 2015). However, penicillin treatment has also been shown to limit genital tract inflammatory lesion formation in *C. muridarum*-infected mice (Dumoux et al., 2013), a finding inconsistent with beta lactam-induced chlamydial stress dependent inflammatory pathogenesis. Additionally, iron deprivation, know to induce the chlamydial stress response (Hogan et al., 2004), reduced *C. pneumoniae*-induced NFκB activation and IL-6 secretion (Visseren et al., 2002), illustrating the potential for non-beta lactam-induced chlamydial stress to limit NFκB-associated inflammation. The variable nature of the reported effects of chlamydial stress on *in vitro* NFκB activation and IL-6 secretion raises the possibility that both chlamydial species and persistence inducer influence the role that chlamydial stress may play in NFκB-mediated inflammation.

As described above, CHX exposure increased nuclear translocation of NFκB in the context of both *C. pecorum* productive infection and during the penicillin-induced *C. pecorum* stress response. Our finding that CHX exposure also increased nuclear translocation/activation of NFκB in the context of TNFa stimulation, as well as chlamydial infection, strongly suggests that the observed CHX-dependent increase in NFκB nuclear translocation/activation upon chlamydial infection is, at least in part, due to effects independent of chlamydiae/chlamydial infection. We, and others, postulate that CHX-induced suppression of host cellular protein synthesis reduces IKB production, allowing increased nuclear transport of activated NFκB. Though CHX exposure is an artificial means of suppressing mammalian protein synthesis, suspension of host cellular protein synthesis could occur in the context of *in vivo* chlamydial infection as well. For example, George et al. recently demonstrated that chlamydial infection activates the unfolded protein response (UPR) in infected host cells. Specifically, chlamydial infection activates the Protein kinase RNA-activated (PKR)-like ER kinase (PERK) pathway (George et al., 2017). Because activation of the PERK pathway during the URP is known to produce a translational blockade in mammalian cells, it seems plausible that *Chlamydia*-induced UPR activation could decrease IKB production and lead to increased NFκB nuclear translocation *in vivo*—at least in chlamydia-infected cells. Furthermore, interferon gamma (IFN-gamma) is a very well-characterized inducer of chlamydial stress and AB formation. Unlike penicillin, which acts by inhibiting chlamydial peptidoglycan synthesis, IFN-gamma induces chlamydial stress by inducing the host cellular indoleamine-pyrrole 2,3-dioxygenase (IDO) enzyme, which reduces cellular L-tryptophan and essentially starves the developing chlamydiae of this amino acid. Interestingly, the human IKB alpha protein contains 3 tryptophan residues, suggesting that its synthesis would also be inhibited in IDO-expressing cells. Therefore, IFN-gamma-mediated IDO induction represents an additional mechanism by which NFκB translocation could be increased in both productively and persistently *Chlamydia*-infected cells. Because IFN-gamma exposure also induces IDO production in uninfected epithelial cells (Taylor and Feng, 1991), this mechanism would also be predicted to increase NFκB translocation in uninfected, bystander cells as well.

The 24 hpi IF microscopic analysis of NFκB localization, though somewhat difficult to interpret since the presence of chlamydial inclusions in the cytoplasm slightly obscured the observation of cytoplasmic NFκB, nevertheless showed, qualitatively, that NFκB was not predominantly nuclear, but both nuclear and cytoplasmic, at 24 hpi in the absence of CHX. This, in addition to the observed 24 hpi *C. pecorum*-dependent cytoplasmic NFκB activation in this study, is in line with a recent report that wild type *C. trachomatis* is capable of retaining HeLa NFκB in the cytoplasm at 20 hpi, while CPAF-deficient *C. trachomatis* induces marked NFκB nuclear translocation at 20 hpi (Patton et al., 2016). These findings together suggest that NFκB activated by chlamydiae may nevertheless be retained, to some degree in the cytoplasm, and may represent a chlamydial mechanism associated with prevention of the anti-chlamydial affects of a continued inflammatory response. It is expected that the chlamydiae, like many other pathogens, have a broad arsenal of mechanisms to subvert the normal inflammatory process important to the elimination of infection. Understanding the induction of inflammation during chlamydial infection may yet inform important therapeutic targets for the prevention or resolution of infection, or for limiting the eventual damage associated with such infections. Furthermore, little is known about the impact of *Chlamydia*-associated inflammation in the absence of overt disease, or the economic implications of such inflammation the context of farmed animals. The role of NFκB in chlamydial inflammation thus continues to merit consideration.

## Author contributions

CL, RS, and NB designed the study. CL performed the experiments. CL, RS, and NB wrote, revised and approved the manuscript.

## Funding

This work was supported by 310030_147026, www.snf.ch.

### Conflict of interest statement

The authors declare that the research was conducted in the absence of any commercial or financial relationships that could be construed as a potential conflict of interest.
