# Supplementary material for: Productive and Penicillin-Stressed Chlamydia pecorum Infection Induces Nuclear Factor Kappa B Activation and Interleukin-6 Secretion In Vitro
Source: Front Cell Infect Microbiol. 2017 May 11;7:180. doi: 10.3389/fcimb.2017.00180 (PMC5425588; doi:10.3389/fcimb.2017.00180)
Supplement: Supplementary file 1 [file Image1.PDF]

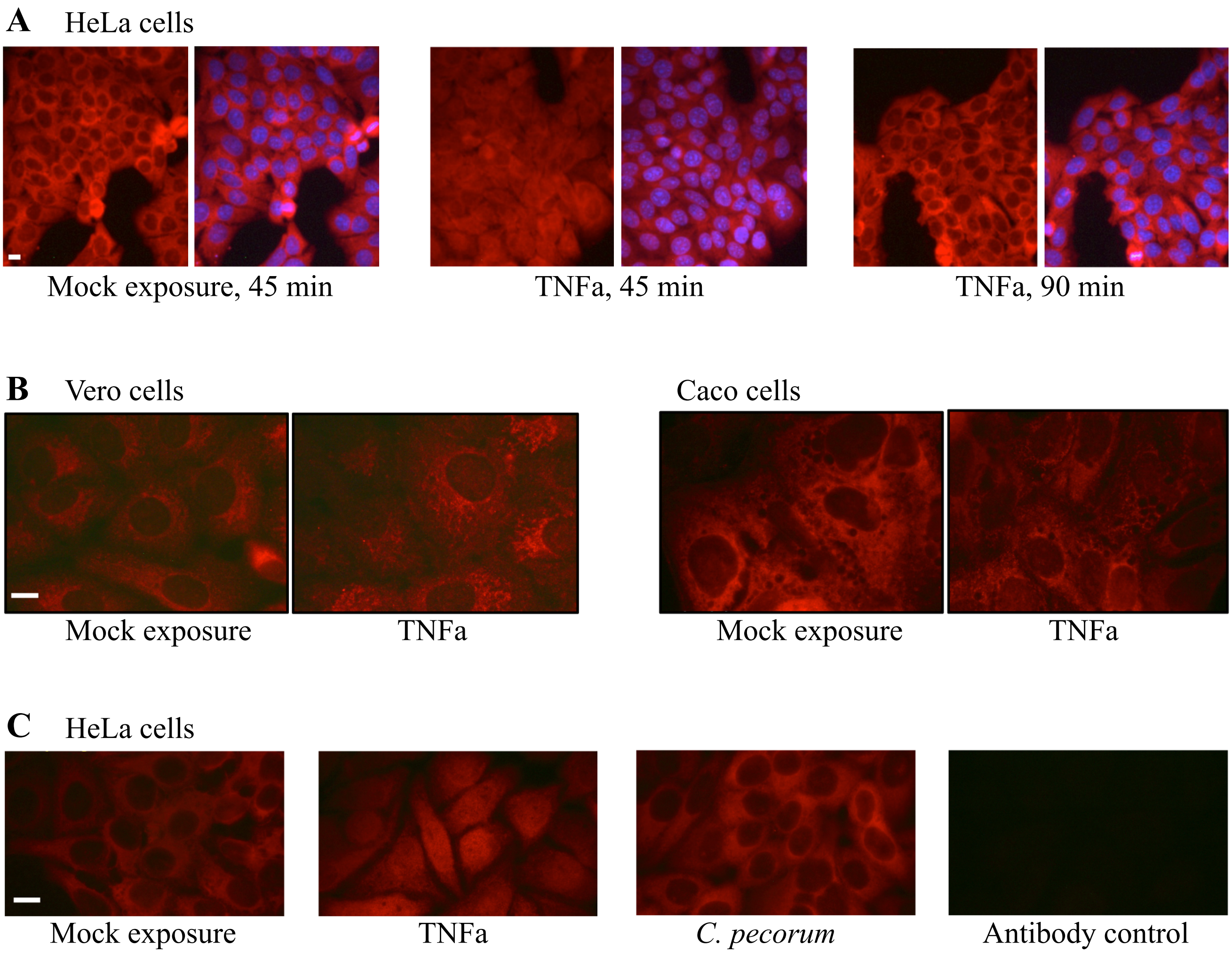

Supplemental Figure 1. **Response of HeLa Cells to TNFa Exposure. (A)** HeLa cells were exposed to TNFa in the culture medium at 20, 50 (shown) or 100 ng/mL and incubated for 45, 60, or 90 minutes (min) before fixing and immunofluorescence microscopy detection of NFkB p65 labeling (red) and DNA (blue) at 200X magnification. Nuclear translocation was observed upon exposure to all TNFa concentrations at 45 min and 60 min (not shown) post exposure. NFkB nuclear translocation returned to levels similar to mock exposure at 90 minutes post exposure. **(B)** Vero and Caco cells were exposed to TNFa in the culture medium as described for HeLa cells in (A). NFkB p65 (red) labeling is shown at 1000X magnification. Nuclear translocation was not observed upon exposure to any TNFa concentrations (100 ng/mL shown) at any time (60 min shown). **(C)** HeLa cells were exposed to *C. pecorum* crude stock (multiplicity of infection = 5 inclusion forming units per cell) in the culture medium, in parallel to mock exposed and 100 ng/mL TNFa exposed cells, for 45 or 60 min (shown) as described in (A). NFkB p65 labeling (red) is shown at 1000X magnification. *C. pecorum* exposure failed to elicit NFkB translocation under these exposure conditions. An antibody control, with no primary NFkB p65 antibody incubation (primary incubation was with antibody diluent alone), but normal secondary antibody incubation was used to confirm specific labeling with the NFkB p65 primary antibody used in this study. Scale bars (A, B, and C) = 10 µm.
