# Supplementary material for: Productive and Penicillin-Stressed Chlamydia pecorum Infection Induces Nuclear Factor Kappa B Activation and Interleukin-6 Secretion In Vitro
Source: Front Cell Infect Microbiol. 2017 May 11;7:180. doi: 10.3389/fcimb.2017.00180 (PMC5425588; doi:10.3389/fcimb.2017.00180)
Supplement: Supplementary file 2 [file Image2.PDF]

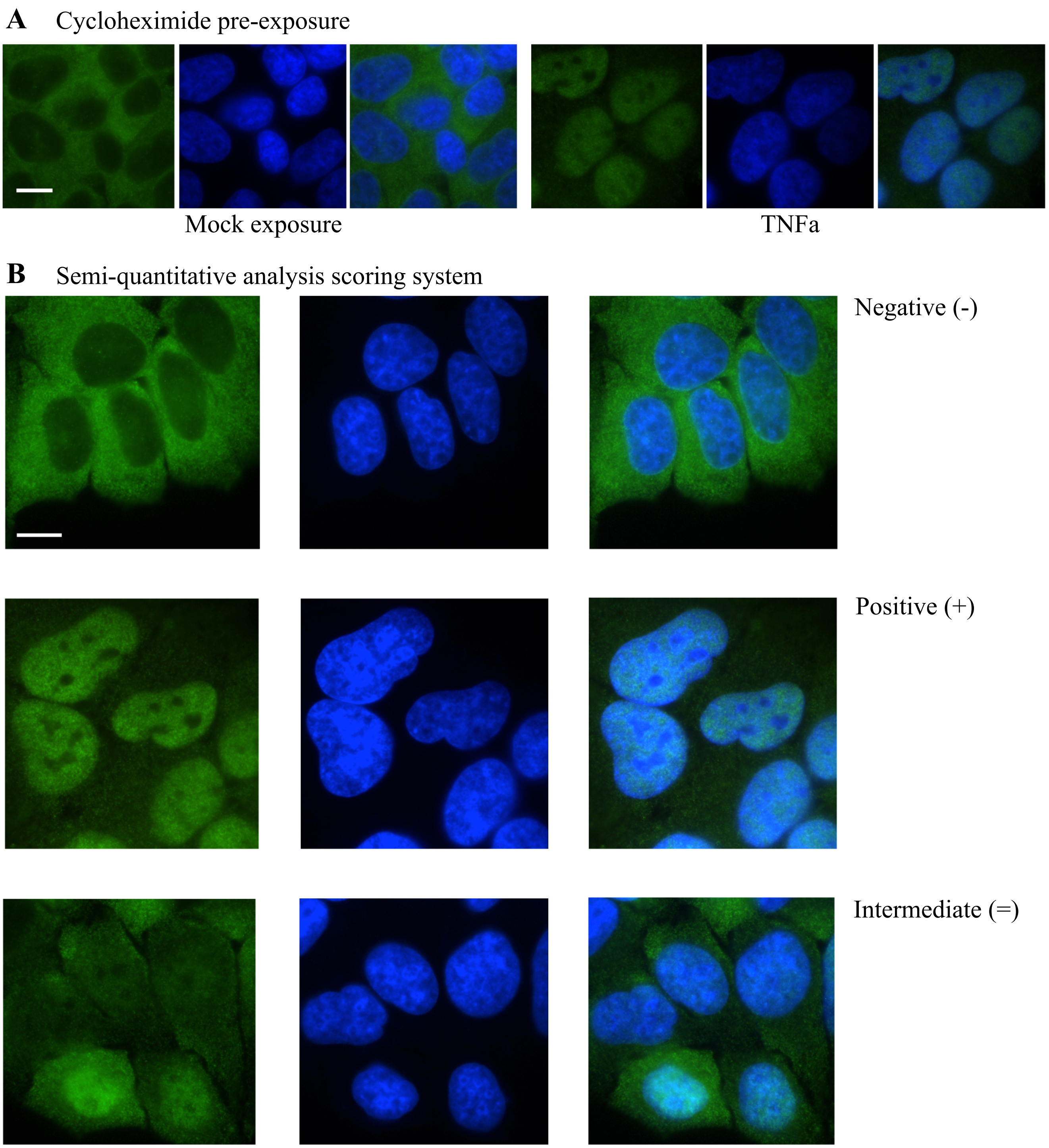

Supplemental Figure 2. Cycloheximide Pre-Exposure Increases the Degree of NFkB Nuclear Translocation, Facilitating Detection of Translocation and Subsequent Semi-Quantitative Analysis. (A) HeLa cells were pre-exposed, for 2 hours, to 1 or 5 (shown) µg/ml cycloheximide in the culture medium, then exposed to 100 ng/mL TNFa in the culture medium for 45 minutes prior to immunofluorescence microscopy detection of NFkB p65 labeling (green) and DNA (blue) at 1000X magnification. Marked nuclear translocation was observed upon pre-exposure to cycloheximide and was stronger at 5 µg/mL. **(B)** Scoring system for semi-quantitative analysis of NFkB labeling, wherein the degree of NFkB nuclear translocation was considered. HeLa mock exposure, 5 µg/mL cycloheximide and 20 ng/mL TNFa exposure, or TNFa exposure alone for 60 minutes were used for the negative, positive and intermediate images shown, respectively. Scale bars (A and B) = 10 µm.
