# Supplementary material for: Productive and Penicillin-Stressed Chlamydia pecorum Infection Induces Nuclear Factor Kappa B Activation and Interleukin-6 Secretion In Vitro
Source: Front Cell Infect Microbiol. 2017 May 11;7:180. doi: 10.3389/fcimb.2017.00180 (PMC5425588; doi:10.3389/fcimb.2017.00180)
Supplement: Supplementary file 3 [file Image3.PDF]

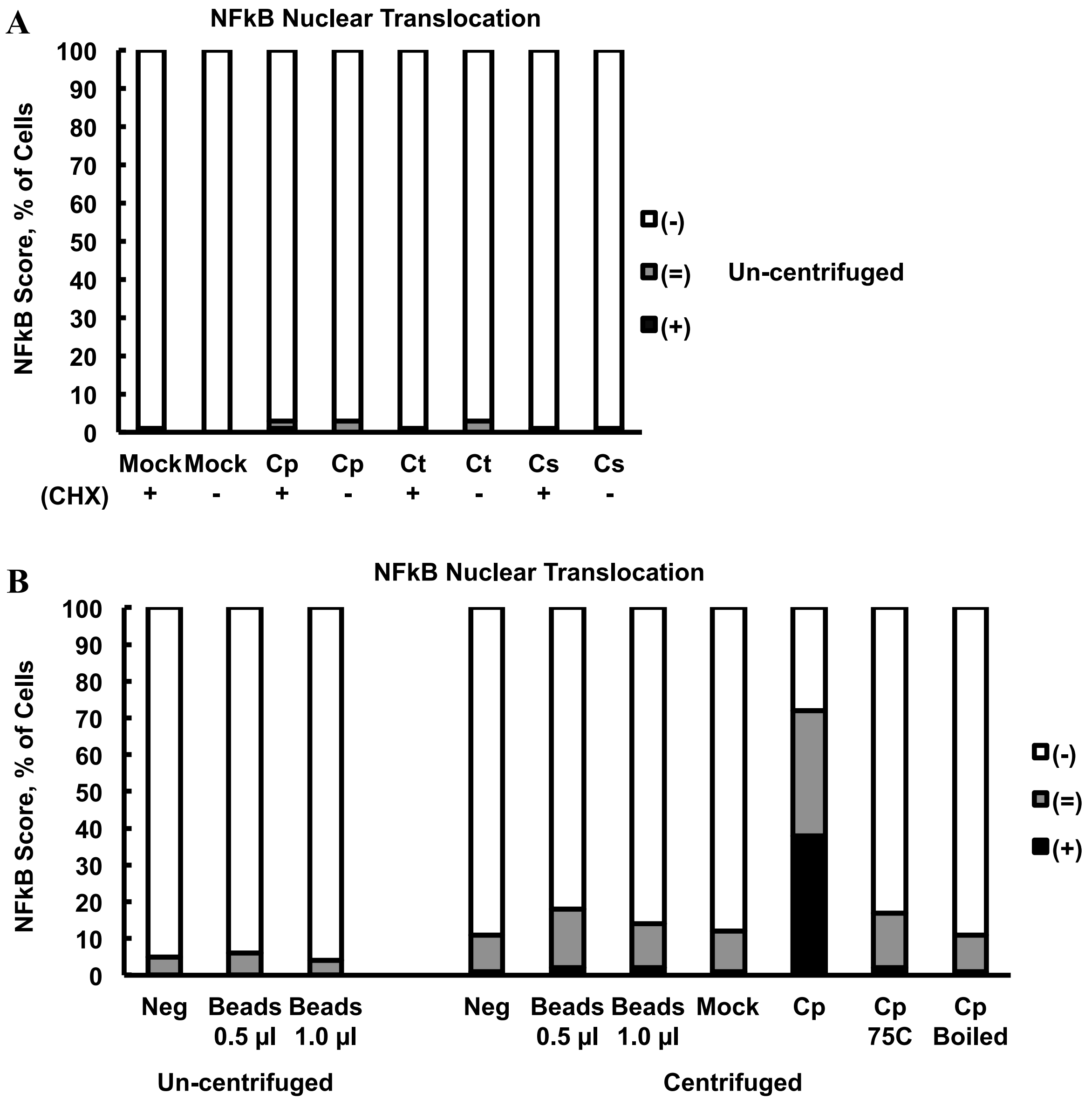

Supplemental Figure 3. ***Chlamydia pecorum*****-Induced Nuclear Factor Kappa B (NFkB) Nuclear Translocation is Centrifugation-Dependent, but Particle Centrifugation Alone Does Not Induce NFkB Nuclear Translocation.** HeLa cells were pre-exposed (+), or not (-), to 5 µg/mL cycloheximide (CHX) for 2 hours (h), infected, with or without centrifugation, with *C. pecorum* (Cp), *C. trachomatis* (Ct), or *C. suis* (Cs) (multiplicity of infection of 5) and incubated for 2 h. **(A)** Semi-quantitative analysis of NFkB nuclear translocation assayed by IF microscopy, wherein 100 cells per group were scored positive (+), intermediate (=) or negative (-) for NFkB nuclear translocation, showed that *Chlamydia* did not induce NFkB nuclear translocation in the absence of centrifugation. **(B)** Semi-quantitative analysis showed that centrifugation-assisted infection with heat treated/inactivated *C. pecorum,* whether boiled or heated at 75°C, was unable to induced the NFkB nuclear translocation observed for non-heat treated/inactivated *C. pecorum*. Non-infectious latex particles (Beads; 0.5 µl/well ≈ 8 particles per cell, 1.0 µl/well ≈ 16 particles per cell), whether centrifuged or not, also failed to induce NFkB nuclear translocation.
