# Supplementary material for: Productive and Penicillin-Stressed Chlamydia pecorum Infection Induces Nuclear Factor Kappa B Activation and Interleukin-6 Secretion In Vitro
Source: Front Cell Infect Microbiol. 2017 May 11;7:180. doi: 10.3389/fcimb.2017.00180 (PMC5425588; doi:10.3389/fcimb.2017.00180)
Supplement: Supplementary file 4 [file Image4.PDF]

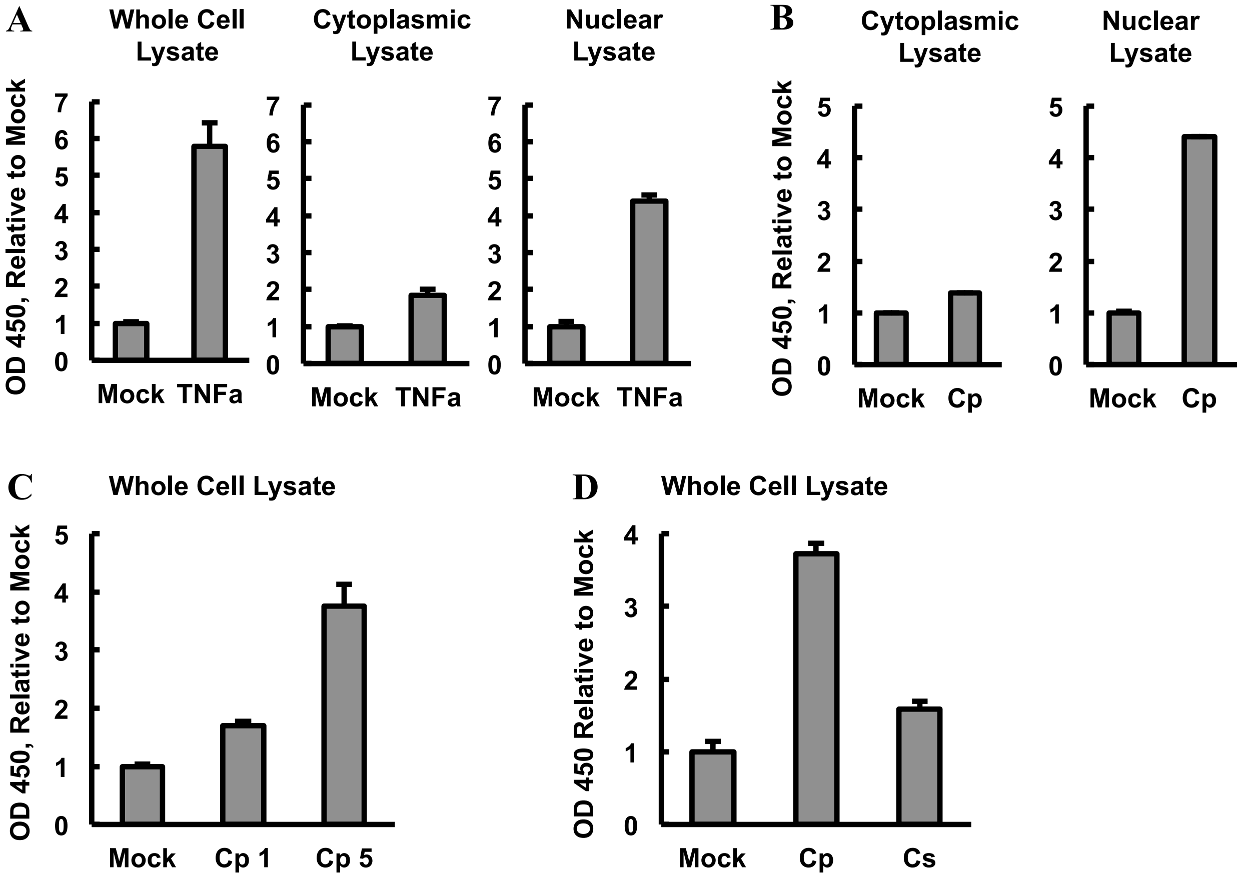

Supplemental Figure 4. *Chlamydia*-Induced Nuclear Factor Kappa B (NFkB) Activation at 2 Hours Post Infection is Largely Associated With the Nucleus, is Dose-Dependent and is Species-Specific. HeLa cells were pre-exposed to 1 µg/mL cycloheximide for 2 hours (h), infected (with centrifugation) with *C. pecorum* (Cp) or *C. suis* (Cs) (multiplicity of infection of 5 unless noted as 1 (Cp1)), or exposed (without centrifugation) to 20 ng/mL tumor necrosis factor alpha (TNFa) and incubated for 2 h. NFkB activation, specifically of subunit p65, was assayed by an ELISA-style assay of whole cell, cytoplasmic or nuclear lysates. **(A)** TNFa exposure induced NFkB activation, mainly associated with nucleus and detectable in the whole cell lysate. *C. pecorum*-dependent NFkB activation was also mainly associated with the nucleus **(B)**, was dose-dependent **(C)** and was more robust than that observed for *C. suis* **(D)**.
