# Supplementary material for: Productive and Penicillin-Stressed Chlamydia pecorum Infection Induces Nuclear Factor Kappa B Activation and Interleukin-6 Secretion In Vitro
Source: Front Cell Infect Microbiol. 2017 May 11;7:180. doi: 10.3389/fcimb.2017.00180 (PMC5425588; doi:10.3389/fcimb.2017.00180)
Supplement: Supplementary file 5 [file Image5.PDF]

A

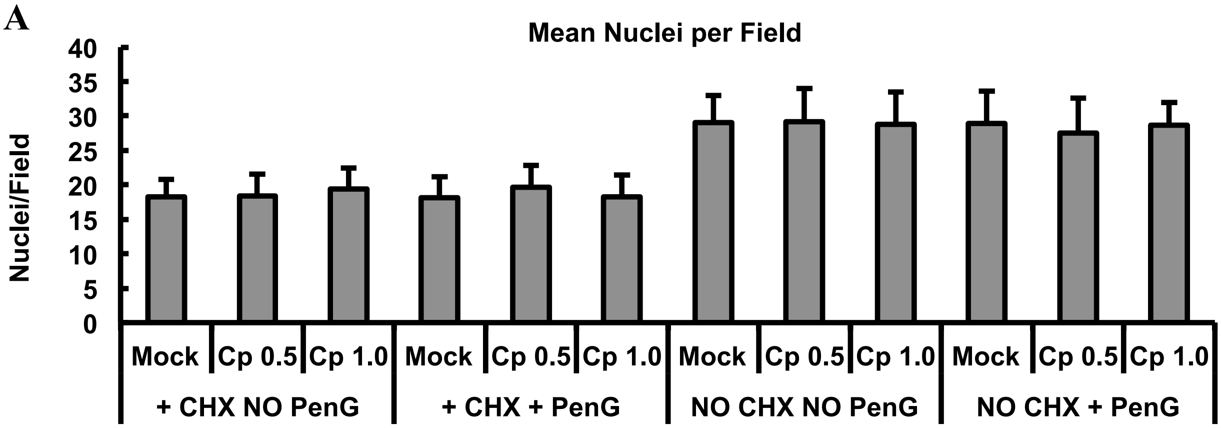

B

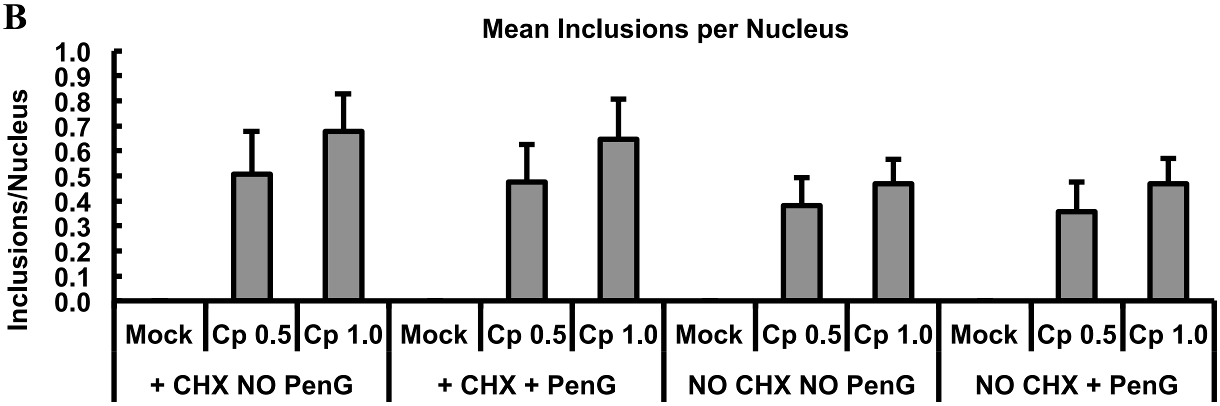

Supplemental Figure 5. *C. pecorum* Infection Does Not Cause Cell Lysis; Penicillin Does Not Prevent Inclusion Formation. HeLa cells were pre-exposed, or not, to 1 µg/mL CHX for 2 hours (h), infected (with centrifugation) with *C. pecorum* (Cp) (multiplicity of infection of 0.5 or 1), and incubated for 24 h with or without PenG. Mean nuclei per field **(A)** and mean inclusions per nucleus **(B)** are shown.
