# Supplementary material for: Productive and Penicillin-Stressed Chlamydia pecorum Infection Induces Nuclear Factor Kappa B Activation and Interleukin-6 Secretion In Vitro
Source: Front Cell Infect Microbiol. 2017 May 11;7:180. doi: 10.3389/fcimb.2017.00180 (PMC5425588; doi:10.3389/fcimb.2017.00180)
Supplement: Supplementary file 6 [file Image6.PDF]

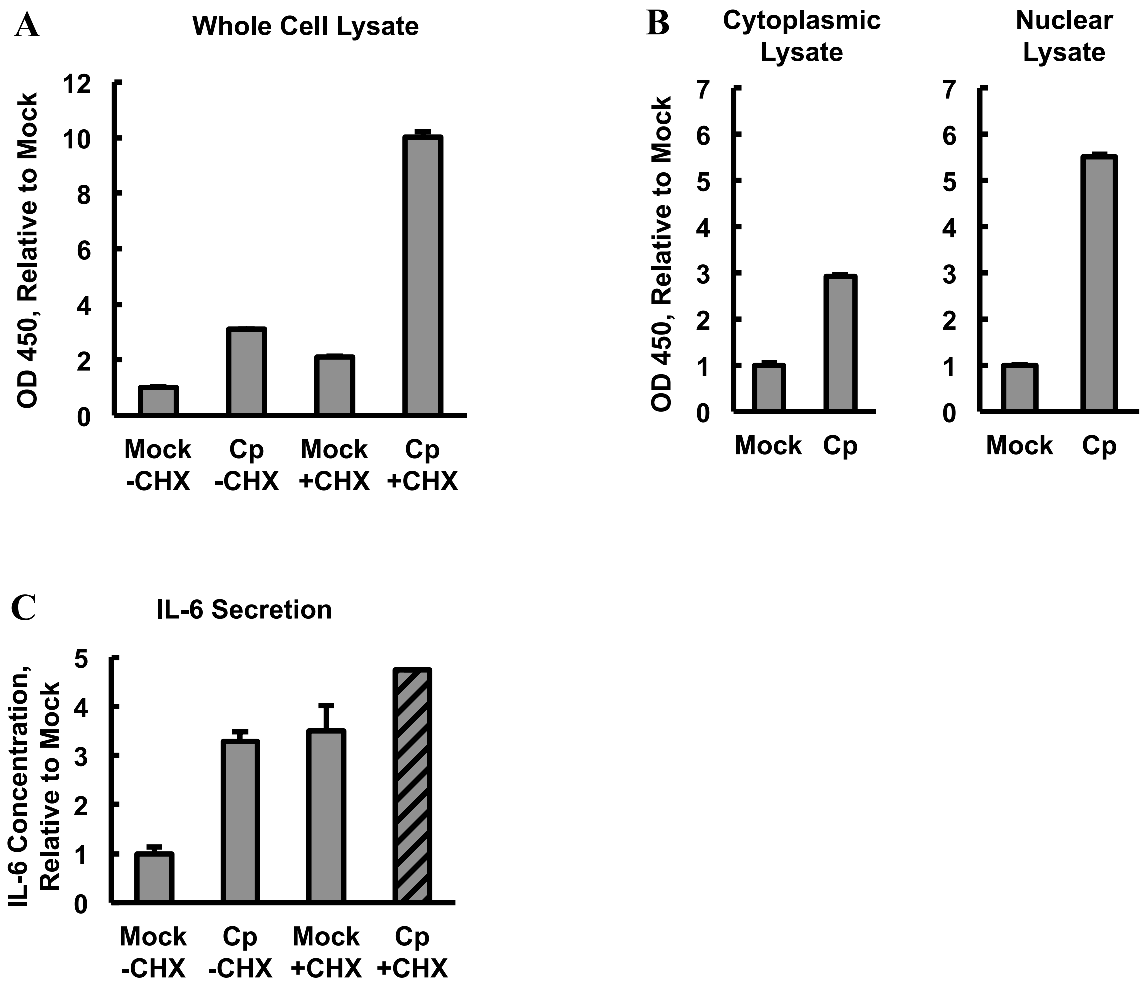

Supplemental Figure 6. *Chlamydia*-Induced Nuclear Factor Kappa B (NFkB) Activation and Interleukin 6 (IL-6) Secretion at 24 Hours Post Infection. HeLa cells were pre-exposed (+) or not (-) to 1 µg/mL cycloheximide (CHX) for 2 hours (h), infected (with centrifugation) with MOI 1 *C. pecorum* (Cp) and incubated until 24 h post infection (hpi). **(A, B)** NFkB activation, specifically of subunit p65, was assayed by an ELISA-style assay of whole cell, cytoplasmic or nuclear lysates. NFkB nuclear translocation was substantial in the absence of CHX and markedly potentiated by CHX exposure. Both cytoplasmic and nuclear lysates of *C. pecorum* infected cells showed marked increase in NFkB activation versus mock-infected cell lysates. **(C)** IL-6 secretion was assayed by ELISA evaluation of cell culture medium and showed that *C. pecorum* substantially induced IL-6 secretion, and this effect was also potentiated by CHX (note that the value shown for the *C. pecorum* +CHX group represents an underestimation due to detection limitations of the assay as performed for the single supplemental experiment (detection range was limited to ≤2099 pg/mL IL-6)).
